# Post-operative delirium in different age groups and subtypes: a systematic review of case reports

**DOI:** 10.3389/fneur.2024.1465681

**Published:** 2024-10-10

**Authors:** Jiaming Guo, Xiaomei Guo, Wei Liu, Aoran Zhou, Jiayi Han, Runxin Yi, Lijuan Dong, Yinhao Zhou

**Affiliations:** ^1^Department of Nursing, Zhongshan Hospital of Traditional Chinese Medicine, Zhongshan, China; ^2^The First School of Clinical Medicine, Xinxiang Medical University, Xinxiang, China; ^3^Department of Clinical Pharmacy, Zhongshan City People's Hospital, Zhongshan, China

**Keywords:** acute confusion, post-operative delirium, age, subtype, systematic review

## Abstract

**Aims:**

To explore the clinical presentations and outcomes among different ages and subtypes of post-operative delirium patients.

**Design:**

Systematic review of Published Cases.

**Methods and data sources:**

We comprehensively searched PubMed, EMBASE, and MEDLINE for published case reports of post-operative delirium up to April 2023. The systematic review has been registered with PROSPERO. Two researchers independently conducted unblinded reviews of the full-text articles.

**Results:**

This study included 116 patients with post-operative delirium. Compared to post-operative delirium patients aged 65 and above, those between 18 and 65 years old have lower rates of a history of hypertension, cardiovascular disease and urinary system disorder comorbidities, as well as higher usage rates of fentanyl analogs and lorazepam. Additionally, these patients exhibit lower incidences of anemia and renal failure, along with a lower mortality rate. Compared to post-operative delirium patients aged 65 and above, those under 18 years old have a higher rate of fentanyl analog usage and a higher incidence of post-operative delirium following neurological surgeries. Among the hypoactive, hyperactive, and mixed subtypes, the reasons for surgery, such as cardiovascular diseases, reproductive system diseases, and neurological disorders, significantly varied among these three subtypes. Furthermore, substance abuse history and medication usage patterns also significantly varied among these three subtypes.

**Conclusions:**

Our investigation has revealed noteworthy insights into post-operative delirium in different patient populations. Notably, age emerged as a pivotal factor. Compared to elderly patients (≥65 years), those aged 18 to 65 demonstrate better prognosis. Additionally, patients younger than 18 years with post-operative delirium have a higher incidence of delirium following neurosurgical procedures compared to those elderly patients. Additionally, a strong association was found between a history of substance abuse and hyperactive delirium. Variations in drug use patterns were observed across different subtypes. Importantly, post-operative delirium patients younger than 18 years, as well as those aged 18 to 65 with mixed-subtype delirium, exhibited similar high mortality rates as elderly patients. This underscores the need for increased attention to post-operative delirium patients under 65 and highlights the necessity of rapid identification and early intervention for these populations at risk of poor outcomes.

**Systematic review registration:**

https://www.crd.york.ac.uk/prospero/display_record.php?ID=CRD42023473383, Identifier [Registration ID: CRD 42023473383].

## 1 Introduction

Post-operative delirium (POD) is a prevalent transient cerebral dysfunction that frequently arises in a significant proportion of older adults (≥65 years) undergoing surgical procedures (1). It is characterized by acute onset and fluctuations in consciousness and cognition, leading to increased morbidity, length of hospital stays, healthcare expenditure, and mortality (2–5). Extensive research has been conducted on post-operative delirium in older populations, highlighting various risk factors, such as high-risk surgical procedures; pain; comorbidity burdens; histories of neurological, cardiac, respiratory, and metabolic diseases; metabolic derangement; and deficient functional baseline (3). Recent studies have found that certain biomarkers may also have predictive value for the occurrence of post-operative delirium, such as the C-reactive protein albumin ratio (6) and serum ferritin (7).

Research on post-operative delirium has focused primarily on adults or elderly patients aged 65 years and older (4, 8–10). Due to the lower incidence and mortality rates (11), studies on the characteristics and prognosis of post-operative delirium in patients aged younger than 65 years are limited. Previous studies have indicated that both children and non-elderly adult patients exhibit atypical symptoms, posing challenges for early diagnosis in these two populations (12). Furthermore, our study revealed that children and nonelderly adult patients often experience prolonged hospital stays (Table 1, Supplementary material 1), which typically translates into increased utilization of healthcare resources and additional medical costs (13, 14). Notably, post-operative delirium patients under the age of 18 often have underdeveloped brain regions related to “post-operative delirium,” which may result in significant differences in their clinical presentation and prognosis compared to other patient groups (15, 16). Therefore, understanding the unique clinical features and potential risk factors for post-operative delirium in children and non-elderly adults is of paramount importance. It assists healthcare professionals in better identifying and managing patients, devising targeted intervention measures, improving patient outcomes, and reducing both hospitalization duration and medical expenses.

**Table 1:** Clinical characteristics and outcomes of adult patients with post-operative delirium.

**Clinical characteristics**	**POD-ALL**	**POD-Y (18 ≤ y < 65)**	**POD-E (y ≥65)**	***p*-value^a^**
Number of patients	116	63	45	
Age (all)	55.71 ± 22.43	45.81 ± 13.52	77.16 ± 7.62	**< 0.001**
Age (female)	55.78 ± 21.60	45.93 ± 12.61	76.71 ± 7.97	**< 0.001**
Age (male)	55.65 ± 23.14	45.70 ± 14.30	77.54 ± 7.27	**< 0.001**
Male	61/115 (53.04%)	33/63 (52.38%)	24/45 (53.33%)	0.922
**Reasons for surgery**				
Skeletal disorders	24/116 (20.69%)	14/63 (22.22%)	9/45 (20.00%)	0.781
Cardiovascular diseases	22/116 (18.97%)	11/63 (17.46%)	9/45 (20.00%)	0.738
Gastrointestinal diseases	23/116 (18.93%)	9/63 (14.29%)	13/45 (28.89%)	0.063
Malignant neoplasm	12/116 (10.34%)	6/63 (9.52%)	6/45 (13.33%)	0.535
Respiratory diseases	7/116 (6.03%)	3/63 (4.76%)	4/45 (8.89%)	0.644
Reproductive system diseases	6/116 (5.17%)	6/63 (9.52%)	0/45 (0.00%)	0.088
Neurological disorders	5/116 (4.31%)	3/63 (4.76%)	0/45 (0.00%)	0.373
Urological disorders	4/116 (3.45%)	1/63 (1.59%)	3/45 (6.67%)	0.389
Others	13/116 (11.21%)	10/63 (15.87%)	1/45 (2.22%)	**0.047**
**Anesthesia**				
Regional	8/94 (8.51%)	4/54 (7.41%)	4/32 (12.50%)	0.688
General	86/94 (91.49%)	49/54 (90.74%)	28/32 (87.50%)	0.912
**Preexisting cognitive dysfunction**	16/109 (14.68%)	9/58 (15.52%)	7/43 (16.28%)	0.917
**Delirium history**	2/108 (1.85%)	2/58 (3.45%)	0/42 (0.00%)	0.334
**Disease history**				
Smoke	9/105 (8.57%)	7/56 (12.50%)	2/41 (4.88%)	0.356
Alcohol	9/105 (8.57%)	8/56 (14.29%)	1/41 (2.44%)	0.103
Drugs (cocaine etc.)	7/105 (6.67%)	7/56 (12.50%)	0/41 (0.00%)	0.051
Common chronic disease	41/105 (39.05%)	14/56 (25.00%)	25/41 (60.98%)	**< 0.001**
Dyslipidemia	8/105 (7.62%)	3/56 (5.36%)	5/41 (12.20%)	0.403
Diabetes	14/105 (13.33%)	5/56 (8.93%)	8/41 (19.51%)	0.118
Hypertension	24/105 (22.86%)	6/56 (10.71%)	17/41 (41.46%)	**< 0.001**
Coronary heart disease	8/105 (7.62%)	2/56 (3.57%)	6/41 (14.63%)	0.113
Asthma	5/105 (4.76%)	2/56 (3.57%)	3/41 (7.32%)	0.719
COPD	5/105 (4.76%)	1/56 (1.79%)	3/41 (7.32%)	0.403
**Intraoperative complications**				
Hypotension	2/59 (3.39%)	1/33 (3.03%)	1/20 (5.00%)	0.617
Hypertension	1/59 (1.69%)	1/33 (3.03%)	0/20 (0.00%)	0.623
Desaturation	0/59 (0.00%)	0/33 (0.00%)	0/20 (0.00%)	-
Blood transfusion	2/59 (3.39%)	0/33 (0.00%)	2/20 (10.00%)	0.138
**Subtypes**				
Hypoactive	38/89 (42.70%)	7/57 (12.28%)	4/31 (12.90%)	1
Hyperactive	63/89 (70.79%)	34/57 (59.65%)	21/31 (67.74%)	0.454
Mixed	15/89 (16.85%)	3/57 (5.26%)	6/31 (19.35%)	0.086
**Time of surgery**				
Mean ± SD min	214.93 ± 125.77	286.10 ± 158.16	192.3 ± 103.76	0.091
**Comorbidities**				
Cardiovascular	39/106 (36.79%)	11/56 (19.64%)	27/43 (62.79%)	**< 0.001**
Gastrointestinal	18/106 (16.98%)	11/56 (19.64%)	7/43 (16.28%)	0.667
Central nervous system	18/106 (16.98%)	9/56 (16.07%)	8/43 (18.60%)	0.740
Respiratory	15/106 (14.15%)	7/56 (12.50%)	7/43 (16.28%)	0.593
Hematological	5/106 (4.72%)	2/56 (3.57%)	3/43 (6.98%)	0.761
Urinary	13/106 (12.26%)	3/56 (5.36%)	10/43 (23.26%)	**0.009**
Mental disorders	22/106 (20.75%)	15/56 (26.79%)	8/43 (18.60%)	0.339
Anxiety	6/106 (5.66%)	5/56 (8.93%)	1/43 (2.33%)	0.347
Depression	7/106 (6.60%)	5/56 (8.93%)	2/43 (4.65%)	0.669
Cancer	16/106 (15.09%)	6/56 (10.71%)	10/43 (23.26%)	0.093
Else	27/106 (25.47%)	16/56 (28.57%)	32/43 (74.42%)	**< 0.001**
**Anesthetic drugs**				
Sevoflurane	18/50 (36.00%)	11/43 (25.58%)	2/12 (16.67%)	0.796
Isoflurane	7/50 (14.00%)	6/43 (13.95%)	1/12 (8.33%)	0.979
Nitrous oxide	8/50 (16.00%)	5/43 (11.63%)	0/12 (0.00%)	0.502
Propofol	29/50 (58.00%)	21/43 (48.84%)	6/12 (50.00%)	0.943
Ketamine	5/50 (10.00%)	4/43 (9.30%)	1/12 (8.33%)	1
Thiopental sodium	3/50 (6.00%)	3/43 (6.98%)	0/12 (0.00%)	0.470
Lidocaine	16/50 (32.00%)	8/43 (18.60%)	5/12 (41.67%)	0.201
Bupivacaine	3/50 (6.00%)	1/43 (2.33%)	1/12 (8.33%)	0.392
**Medicine**				
Fentanyl analogs	40/102 (39.22%)	27/62 (43.55%)	8/36 (22.22%)	**0.034**
Fentanyl	18/102 (17.65%)	9/62 (14.52%)	6/36 (16.67%)	0.776
Sufentanil	5/102 (4.90%)	4/62 (6.45%)	1/36 (2.78%)	0.748
Remifentanil	5/102 (4.90%)	5/62 (8.06%)	0/36 (0.00%)	0.148
Morphine	7/102 (6.86%)	8/62 (12.90%)	10/36 (27.78%)	0.067
Hydrocodone	8/102 (7.84%)	5/62 (8.06%)	3/36 (8.33%)	1
Succinylcholine	7/102 (6.86%)	7/62 (11.29%)	0/36 (0.00%)	0.092
Vecuronium	5/102 (4.90%)	5/62 (8.06%)	0/36 (0.00%)	0.080
Rocuronium	9/102 (8.82%)	8/62 (12.90%)	1/36 (2.78%)	0.190
Benzodiazepines	49/102 (48.04%)	33/62 (53.23%)	11/36 (30.56%)	0.569
Lorazepam	16/102 (15.69%)	13/62 (20.97%)	2/36 (5.56%)	**0.041**
Midazolam	30/102 (29.41%)	20/62 (32.26%)	6/36(16.67%)	0.092
Dexmedetomidine	10/102 (9.80%)	8/62 (12.90%)	2/36 (5.56%)	0.417
Diphenhydramine	6/102 (5.88%)	4/62 (6.45%)	2/36 (5.56%)	1
Chlorpromazine	5/102 (4.90%)	4/62 (6.45%)	1/36 (2.78%)	0.748
Flupentixol	31/102 (30.39%)	19/62 (30.65%)	12/36 (33.33%)	0.783
Quetiapine	10/102 (9.80%)	6/62 (9.68%)	4/36 (11.11%)	1
Olanzapine	7/102 (6.86%)	4/62 (6.45%)	2/36 (5.56%)	1
NSAIDs	20/102 (19.61%)	10/62 (16.13%)	9/36 (25.00%)	0.284
Aspirin	7/102 (6.86%)	2/62 (3.23%)	5/36 (13.89%)	0.117
Atropine	10/102 (9.80%)	9/62 (14.52%)	1/36 (2.78%)	0.132
Neostigmine	9/102 (8.82%)	7/62 (11.29%)	2/36 (5.56%)	0.559
Glucocorticoids	15/102 (14.71%)	11/62 (17.74%)	4/36 (11.11%)	0.379
Furosemide	7/102 (6.86%)	4/62 (6.45%)	3/36 (8.33%)	1
Catecholamines	16/102 (15.69%)	4/62 (6.45%)	6/36 (16.67%)	0.206
Epinephrine	7/102 (6.86%)	4/62 (6.45%)	2/36 (5.56%)	1
Norepinephrine	5/102 (4.90%)	0/62 (0.00%)	5/36 (13.89%)	**0.011**
Dopamine	4/102 (3.92%)	3/62 (4.84%)	1/36 (2.78%)	1
Statins	5/102 (4.90%)	2/62 (3.23%)	3/36 (8.33%)	0.528
Ondansetron	9/102 (8.82%)	5/62 (8.06%)	2/36 (5.56%)	0.954
Antibiotics	23/102 (22.55%)	12/62 (19.35%)	11/36 (30.56%)	0.207
**Adverse events**				
Post-operative hypotension	6/104 (5.77%)	3/55 (5.45%)	3/41 (7.32%)	1
Post-operative hypertension	6/104 (5.77%)	3/55 (5.45%)	3/41 (7.32%)	1
Arrhythmia	18/104 (17.31%)	9/55 (16.36%)	9/41 (21.95%)	0.488
Anemia	6/104 (5.77%)	0/55(0.00%)	6/41 (14.63%)	**0.012**
Kidney failure	5/104 (4.81%)	0/55 (0.00%)	5/41 (12.20%)	**0.028**
Else	50/104 (48.08%)	23/55 (41.82%)	26/41 (63.41%)	**0.036**
**ICU**	32/98 (32.65%)	17/49 (34.69%)	14/41 (34.15%)	0.957
**Mechanical ventilation**	33/98 (33.67%)	14/49 (28.57%)	16/41 (39.02%)	0.295
**Days of hospitalization**				
Mean ± SD day	29.80 ± 43.96	37.00 ± 55.44	20.08 ± 18.97	0.888
**Recurrence**	17/116 (14.66%)	6/63 (9.52%)	10/45 (22.22%)	0.067
**Follow-up**	28/116 (24.14%)	19/63 (30.16%)	8/45 (17.78%)	0.143
**Outcome**				
Death (all)	11/113 (9.73%)	1/61(1.64%)	9/44 (20.45%)	**0.004**
Death (female)	4/52 (7.69%)	1/61 (1.64%)	3/20 (15.00%)	**0.045**
Death (male)	7/61 (11.48%)	0/61 (0.00%)	6/24 (25.00%)	**< 0.001**

POD, post-operative delirium; y, year; COPD, chronic obstructive pulmonary disease; SD, Standard Deviation; min, minutes; NSAIDs, non-steroidal anti-inflammatory drugs; ICU, intensive care unit. ^**a**^Means comparison between POD-Y group and POD-E group using Chi-Squared test. The bold values refer to statistical significance (*P* < 0.05).

Delirium is currently defined by three subtypes, hyperactive, hypoactive and mixed, according to the Diagnostic and Statistical Manual of Mental Disorders 5th edition (17). In patients, hyperactive delirium is characterized by anxiety, agitation, disturbance, and excessive excitability. Hypoactive delirium, on the other hand, is typically characterized by apathy, severe difficulty concentrating, and reduced verbal communication. Mixed delirium refers to patients who may exhibit both hyperactive and hypoactive features at different times (18). A systematic review from 2018 revealed that the hypoactive subtype of delirium patients in the intensive care unit had the highest pooled incidence (11%) and prevalence (17%) and was the most common subtype (19). Furthermore, hypoactive and mixed subtypes of post-operative delirium often led to worse outcomes and consume more healthcare resources (20). Various pathophysiological mechanisms may account for the emergence of delirium, but the mechanisms of these different subtypes are still not fully understood. However, comprehensive systematic studies summarizing the clinical course of different subtypes of patients are lacking to aid in understanding their pathophysiological mechanisms and establishing targeted treatment management.

Therefore, this study will explore the differences in clinical presentations and outcomes among post-operative delirium patients across different age groups and delirium subtypes. We divided the patients into three age groups: POD-J (age < 18), POD-Y (age ≥ 18 and < 65), and POD-E (age ≥ 65). Further, we will compare the clinical characteristics and outcomes of post-operative delirium patients in the POD-J and POD-Y groups with the more common and relatively easily identifiable elderly post-operative delirium patient (POD-E group). This comparison will help clinicians better understand the differing clinical manifestations and prognosis between patients in the POD-J and POD-Y groups vs. those in the POD-E group (aged ≥ 65). This knowledge will assist clinicians in more rapidly identifying these potentially dangerous but often insidious cases, allowing for the early implementation of appropriate interventions to improve the prognosis of post-operative delirium patients. In addition, we classified patients into three subtypes: hyperactive, hypoactive, and mixed subtypes. We will further compare the clinical manifestations and outcomes among these three subtypes of post-operative delirium patients, which will enhance our understanding of the different subtypes, including their triggers, pathogenesis, and pathophysiological mechanisms. This knowledge will also inform the development of effective prevention strategies and interventions.

## 2 Methods

### 2.1 Data sources and searches

We used the following keywords, post-operative delirium, post-operative cognitive dysfunction, POD, POCD, and case reports, to systematically search the PubMed, EMBASE, and MEDLINE databases from their inception until April 2023. We aimed to identify and analyze all relevant case reports documenting the occurrence of post-operative delirium. The search terms used for PubMed and MEDLINE were as follows: “[emergence delirium”[MeSH Terms] OR “emergence”[All Fields] AND “delirium”[All Fields]) OR “emergence delirium”[All Fields] OR “post-operative”[All Fields] AND “delirium”[All Fields]) OR “post-operative delirium”[All Fields] OR “POD”[All Fields] OR (“post-operative cognitive complications”[MeSH Terms] OR (“post-operative”[All Fields] AND “cognitive”[All Fields] AND “complications”[All Fields]) OR “post-operative cognitive complications”[All Fields] OR (“post-operative”[All Fields] AND “cognitive”[All Fields] AND “dysfunction”[All Fields]) OR “post-operative cognitive dysfunction”[All Fields]) OR “POCD”[All Fields]) AND “case reports”[Publication Type] OR “case report”[All Fields]). The search details of the EMBASE query were as follows: “post-operative delirium or POD or post-operative cognitive dysfunction or POCD” and “case report.”

### 2.2 Inclusion and exclusion criteria

The inclusion criteria were as follows: (I) the type of article was a case report or case series; (II) the study was conducted within an inpatient hospital setting, and patients had to be diagnosed with post-operative delirium; (III) the case details needed to encompass one or more of the following elements: age, sex, subtypes, reasons for surgery, disease history, comorbidities, medicine, outcome, etc.; and (IV) the full text of the articles was accessible. In alignment with the exclusion criteria, conference abstracts, non-English and Chinese literature, meta-analyses and systematic reviews, reviews, comments, and unrelated studies were excluded.

### 2.3 Data selection and extraction

**Figure 1:**
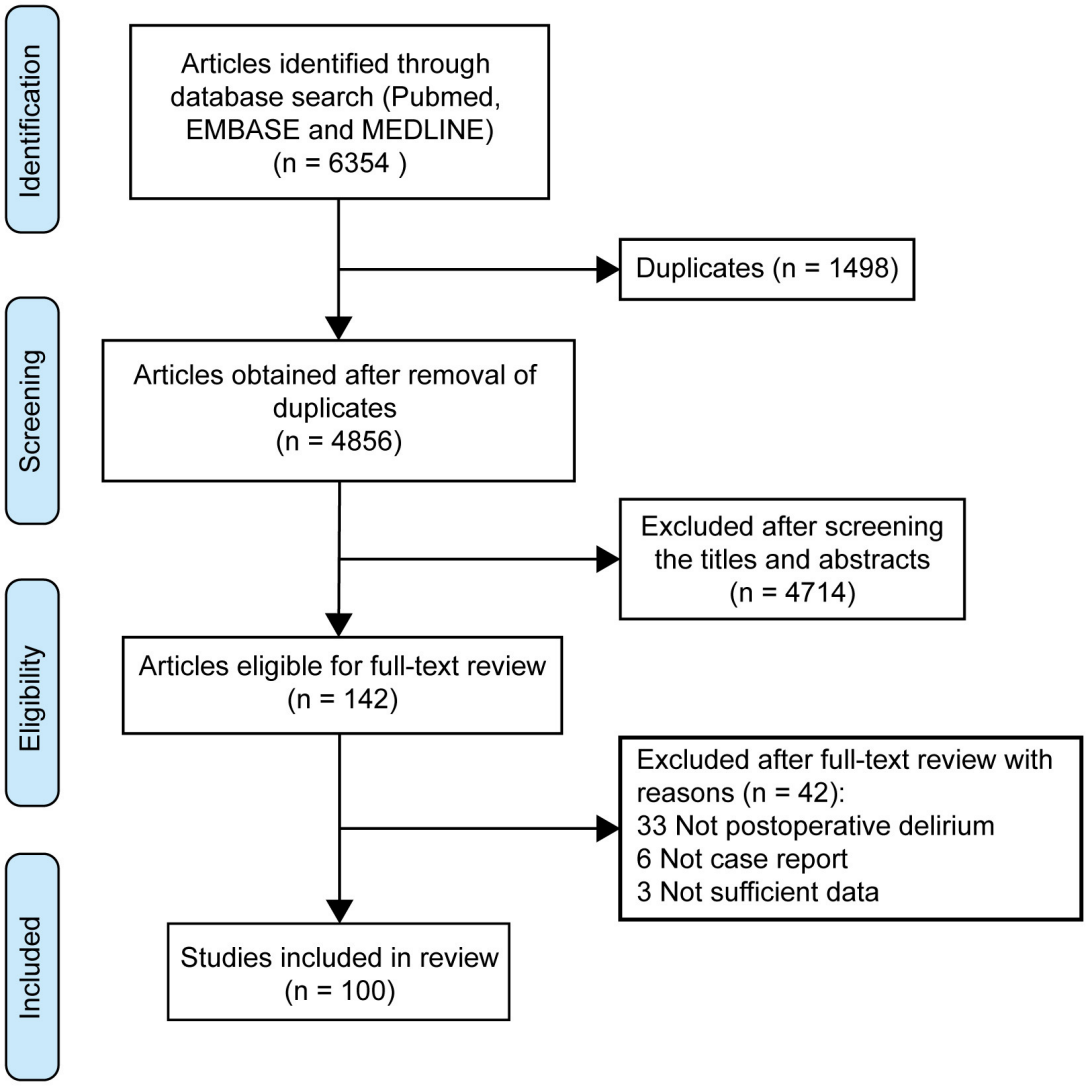
PRISMA flow diagram showing the selection process for patients with post-operative delirium.

Two researchers independently conducted unblinded reviews of the full-text articles and abstracts. A total of 6,354 articles were identified through keyword searches of the databases. After a manual screening process, 6,254 articles were excluded based on the selection criteria. The details of the selection process are presented in Figure 1. From the remaining 100 articles (including 116 patients), various details were extracted, including the publication year, patient demographics (sex, age, etc.), reasons for surgery, anesthesia method, medical history, intraoperative complications, subtypes of post-operative delirium, comorbidities, anesthetic drugs, medication, adverse events, follow-up, and clinical outcomes. The articles describing cases of post-operative delirium resulting from different causes and the details of the clinical characteristics and outcomes of these patients were systematically extracted and documented in the Supplementary material 1.

### 2.4 Quality assessment

Two independent reviewers evaluated the quality of the studies by utilizing the Joanna Briggs Institute (JBI) Critical Appraisal tools for Case Reports (21). The JBI tool assessed the quality of case reports across eight criteria, such as clear identification of the patient's condition, detailed information on treatment interventions, and appropriate follow-up. Each criterion is rated as “Yes” or “No.” The more “Yes” responses, the higher the methodological quality of the case report and the more reliable the evidence provided. Each of the eight criteria in the JBI assessment tool is scored 1 point, with a total score of eight points. The results of the quality assessment are detailed in the Supplementary material 3.

### 2.5 Statistical analysis

We conducted a statistical analysis of the case-derived data. Continuous variables are presented as the mean and standard deviation. For comparing continuous variables, a two-tailed unpaired *t* test was used. Comparisons of the data between different cohorts were conducted using Pearson's chi-square test or Fisher's exact test. A two-sided *p* value < 0.05 indicated statistical significance. IBM SPSS Statistics 27.0.1 was used for all the statistical analyses. Furthermore, GraphPad Prism 8.0.1 was used to construct a heatmap and volcano plot, enhancing the visual representation of the data.

### 2.6 Guidelines statement

The work has been reported in line with PRISMA (Preferred Reporting Items for Systematic Reviews and Meta-Analyses) (22).

## 3 Results

### 3.1 Quality assessment of case reports

One hundred case reports were included in review and the quality assessment, conducted using the Joanna Briggs Institute Critical Appraisal tools for Case Reports, demonstrated that 86 studies scored more than 7, 7 studies scored 6, 4 studies scored 5, 2 studies scored 4, and only one study scored 3. The average JBI score for all included studies was 7.34 (see Supplementary material 3).

### 3.2 Overview of all patients

A total of 116 patients with post-operative delirium (POD-ALL) were described in the 100 studies, and their clinical characteristics and outcomes are detailed in Table 1. Of these patients, 61 (53.04%) were male, with an average age of 55.71 ± 22.43 years. The most common reason for surgery was skeletal disorders (20.69%), followed by cardiovascular diseases (18.97%) and gastrointestinal diseases (18.93%). Eighty-six patients (91.49%) received general anesthesia, and 8 patients (8.51%) received regional anesthesia. Sixteen of the patients (14.68%) had preexisting cognitive dysfunction, and 2 patients (1.85%) had a history of delirium. The majority of these patients had a history of common chronic diseases (39.05%), including hypertension (22.86%), diabetes (13.33%), dyslipidemia (7.62%), and coronary heart disease (7.62%); asthma (4.76%); chronic obstructive pulmonary disease (4.76%); smoking (8.57%); alcohol consumption (8.57%); and drugs (6.67%). The most frequent comorbidities were cardiovascular diseases (36.79%), followed by mental disorders (20.75%), including anxiety (5.66%), and depression (6.60%). The rates of intraoperative complications, including hypotension (3.39%), hypertension (1.69%), and blood transfusion (3.39%), were low, and the average duration of surgery was 214.93 min, with a standard deviation of 125.77 min. Additionally, 38 patients (42.70%) presented as hypoactive, 63 (70.79%) as hyperactive and 15 (16.85%) as mixed. Commonly used anesthetics included propofol (58.00%), sevoflurane (36.00%), and lidocaine (32.00%). The medicines that patients took most often were benzodiazepines (48.04%), including lorazepam (15.69%) and midazolam (29.41%); fentanyl analogs (39.22%), including fentanyl (17.65%), sufentanil (4.90%), and remifentanil (4.90%); flupentixol (30.39%); antibiotics (22.55%); and nonsteroidal anti-inflammatory drugs (19.61%). Remarkably, there was a high incidence of mechanical ventilation (33.67%) and admission to the intensive care unit (32.65%). The average length of hospitalization was 29.80 days, with a standard deviation of 43.96 days. A portion of the patients experienced recurrence (14.66%), and 11 patients (9.73%) died−4 females (7.69%), and 7 males (11.48%).

### 3.3 Clinical features and prognosis of patients with post-operative delirium across different age groups

Among patients aged 65 and older, there is a high incidence of post-operative delirium and poor prognosis, which urgently deserve our attention. However, we always overlook the identification of clinical characteristics and investigations of related prognoses in patients younger than 65 years (the POD-J and POD-Y groups). Tables 1, 2 display the clinical characteristics and outcomes of post-operative delirium patients in the POD-J, POD-Y, and POD-E cohorts.

**Table 2:** Clinical characteristics and outcomes of post-operative delirium patients under 18.

**Clinical characteristics**	**POD-J** **(y < 18)**	**POD-E** **(y ≥65)**	***p*-value^a^**
Number of patients	7	45	
Age (all)	6.94 ± 5.36	77.16 ± 7.62	**< 0.001**
Age (female)	7.67 ± 3.68	76.71 ± 7.97	**< 0.001**
Age (male)	6.40 ± 6.28	77.54 ± 7.27	**< 0.001**
Male	4/7 (57.15%)	24/45 (53.33%)	1
**Reasons for surgery**			
Neurological disorders	2/7 (28.57%)	0/45 (0.00%)	**0.016**
Others	5/7 (71.43%)	1/45 (2.22%)	**< 0.001**
**Anesthesia**			
General	7/7 (100.00%)	28/32 (87.50%)	0.437
**Subtypes**			
Hyperactive	5/7 (71.43%)	21/31 (67.74%)	1
Mixed	2/7 (28.57%)	6/31 (19.35%)	0.978
**Anesthetic drugs**			
Sevoflurane	5/6 (83.33%)	2/12 (16.67%)	**0.026**
Isoflurane	1/6 (16.67%)	1/12 (8.33%)	0.569
Nitrous oxide	3/6 (50.00%)	0/12 (0.00%)	**0.044**
Propofol	2/6 (33.33%)	6/12 (50.00%)	0.867
Lidocaine	3/6 (50.00%)	5/12 (41.67%)	1
Bupivacaine	1/6 (16.67%)	1/12 (8.33%)	0.569
**Medicine**			
Fentanyl analogs	5/7 (71.43%)	8/36 (22.22%)	**0.032**
Fentanyl	3/7 (42.86%)	6/36 (16.67%)	0.293
Morphine	3/7 (42.86%)	10/36 (27.78%)	0.730
Benzodiazepines	5/7 (71.43%)	11/36 (30.56%)	0.105
Lorazepam	1/7 (14.29%)	2/36 (5.56%)	0.421
Midazolam	4/7 (57.14%)	6/36 (16.67%)	0.067
Olanzapine	1/7 (14.29%)	2/36 (5.56%)	0.421
NSAIDs	1/7 (14.29%)	9/36 (25.00%)	0.900
Epinephrine	1/7 (14.29%)	2/36 (5.56%)	0.421
Ondansetron	2/7 (28.57%)	2/36 (5.56%)	0.118
**Adverse Events**			
Abdominal pain	1/7 (14.29%)	1/41 (2.44%)	0.273
Heart failure	1/7 (14.29%)	3/41 (7.32%)	0.480
**ICU**	1/7 (14.29%)	14/41 (34.15%)	0.544
**Mechanical ventilation**	1/7 (14.29%)	16/41 (39.02%)	0.402
**Recurrence**	1/7 (14.29%)	10/45 (22.22%)	1
**Outcome**			
Death (all)	1/7 (14.29%)	9/44 (20.45%)	1
Death (female)	0/3 (0.00%)	3/20 (15.00%)	0.644
Death (male)	1/4 (25.00%)	6/24 (25.00%)	1

POD, post-operative delirium; y, year; NSAIDs, non-steroidal anti-inflammatory drugs; ICU, intensive care unit. ^a^Means comparison between POD-J group and POD-E group using Chi-Squared test. The bold values refer to statistical significance (*P* < 0.05).

**Figure 2:**
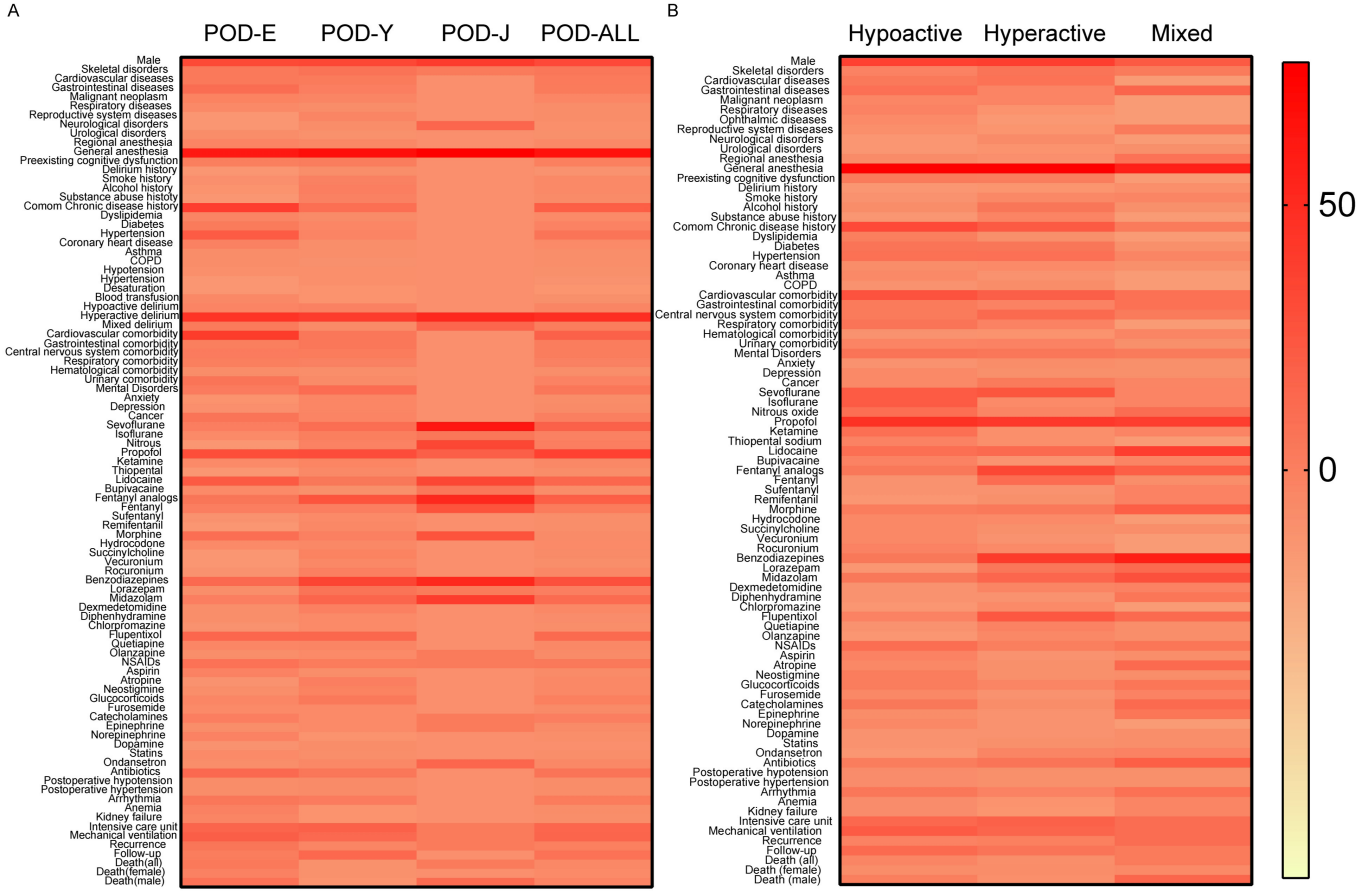
Distribution and variation of various clinical parameters in the respective cohorts. This figure displays data from Tables 1–3. The depth of color in each cell indicates the proportion of the row label in relation to its respective column label (different cohorts). The color spectrum on the far right illustrates the relationship between the proportion size and the color depth. **(A)** Data for different age groups and **(B)** data for three post-operative delirium subtypes. NSAIDs, non-steroidal anti-inflammatory drugs; COPD, chronic obstructive pulmonary disease.

**Figure 3:**
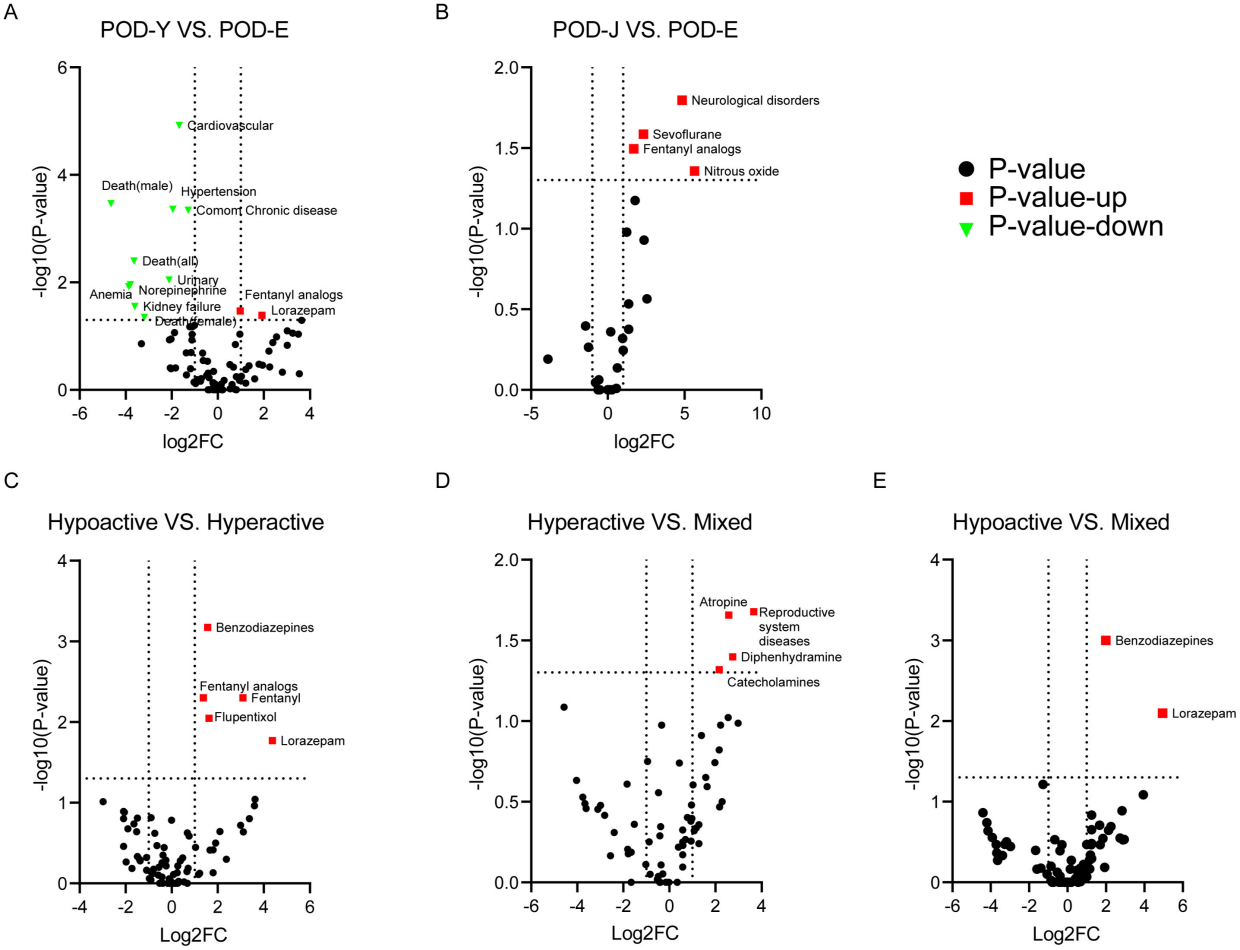
Relationships between changes in various clinical parameters and their significance. The volcano plot illustrates the relationship between changes in various clinical parameters and their significance between the two cohorts. The color of the points represents the significance level, with red indicating upregulation, green indicating downregulation, and black indicating no significant difference. **(A, B)** Relationships between clinical parameter changes and their significance in comparison to those in the POD-E cohort for the POD-Y and POD-J cohorts. **(C–E)** Relationships between clinical parameter changes and their significance according to the post-operative delirium subtypes.

Sixty-three patients were included in the POD-Y group; the average age was 45.81 ± 13.52 years, and 33 patients (52.38%) were men. For the POD-Y group, the most prevalent reason for surgery was skeletal disorders (22.22%), followed by cardiovascular diseases (17.46%) and gastrointestinal diseases (14.29%). Forty-nine patients (90.74%) in the POD-Y group received general anesthesia, and 4 patients (7.41%) received regional anesthesia. Of all patients in the POD-Y cohort, 9 patients (15.52%) had preexisting cognitive dysfunction, and 2 patients (3.45%) had a history of delirium. Common chronic diseases (25.00%) constituted a majority of the disease history in the POD-Y group and included hypertension (10.71%), diabetes (8.93%), dyslipidemia (5.36%), coronary heart disease (3.57%), asthma (3.57%), chronic obstructive pulmonary disease (1.79%), alcohol (14.29%), smoking (12.50%), and drugs (12.50%). In the POD-Y group, the most common comorbidities were mental disorders (26.79%), including anxiety (8.93%) and depression (8.93%), followed by cardiovascular diseases (19.64%), gastrointestinal diseases (19.64%), central nervous system diseases (16.07%), and respiratory diseases (12.50%). The rate of intraoperative complications, including hypotension (3.03%) and hypertension (3.03%), in the POD-Y group was low, and the average duration of surgery was 286.10 min, with a standard deviation of 158.16 min. Additionally, 7 patients (12.28%) in the POD-Y group presented with hypoactivity, 34 (59.65%) with hyperactivity and 3 (5.26%) with mixed symptoms. Propofol (48.84%), sevoflurane (25.58%), and lidocaine (18.60%) were frequently used for anesthesia in the POD-Y group. The medicines that the POD-Y group took most commonly were benzodiazepines (53.23%), which included lorazepam (20.97%) and midazolam (32.26%), followed by fentanyl analogs (43.55%), fentanyl (14.52%), remifentanil (8.06%), sufentanil (6.45%), flupentixol (30.65%), antibiotics (19.35%), and glucocorticoids (17.74%). The POD-Y group also exhibited a high incidence of mechanical ventilation (28.57%) and ICU admission (34.69%). The average length of hospitalization in the POD-Y group was 37.00 days, with a standard deviation of 55.44 days. A portion of the patients experienced recurrence (9.52%), and 1 patient (1.64%) died—including 1 female (1.64%) patient. The clinical characteristics and outcomes of post-operative delirium patients aged 65 years and older (POD-E) are presented in Table 1. We subsequently compared the data of patients aged 18 to 64 (POD-Y) with those of patients in the POD-E group and found that the clinical characteristics and prognoses of the patients in the two cohorts differed. There were significant differences in age between the two cohorts (45.81 ± 13.52 vs. 77.16 ± 7.62, *P* < 0.001) and between females (45.93 ± 12.61 vs. 76.71 ± 7.97, *P* < 0.001) and males (45.70 ± 14.30 vs. 77.54 ± 7.27, *P* < 0.001) within each cohort. Moreover, common chronic diseases (25.00% vs. 60.98%, *P* < 0.001) were less common in the POD-Y group than in the POD-E group, especially hypertension (10.71% vs. 41.46%, *P* < 0.001). Among all the comorbidities, cardiovascular diseases (19.64% vs. 62.79%, *P* < 0.001) and urinary diseases (5.36% vs. 23.26%, *P* = 0.009) were less prevalent in the POD-Y group than in the POD-E group. In addition, the rates of fentanyl analog (43.55% vs. 22.22%, *P* = 0.034) and lorazepam (20.97% vs. 5.56%, *P* = 0.041) use were greater in the POD-Y group than in the POD-E group; however, the rate of norepinephrine (0.00% vs. 13.89%, *P* = 0.011) use was lower. The incidence of adverse events, including anemia (0.00% vs. 14.63%, *P* = 0.012) and kidney failure (0.00% vs. 12.20%, *P* = 0.028), was lower in the POD-Y group than in the POD-E group. Furthermore, the overall in-hospital mortality rate of POD-Y patients was lower than that of POD-E patients (1.64% vs. 20.45%, *P* = 0.004). Additionally, the mortality rates of male (0.00% vs. 25.00%, *P* < 0.001) and female (1.64% vs. 15.00%, *P* = 0.045) POD-Y patients were lower than those of POD-E patients. Additionally, using a heatmap, Figure 2A shows the data in Table 1, and a volcano plot was generated to show the differences and significance of the differences in clinical characteristics and outcomes between the POD-Y group and the POD-E group (Figure 3A).

The POD-J group consisted of 7 patients, with an average age of 6.94 ± 5.36 years, and 4 patients (57.15%) were male. The major reason for surgery in the POD-J group was neurological disorders (28.57%), and all patients underwent surgery under general anesthesia. During post-operative delirium, 5 patients in the POD-J group were hyperactive (71.43%), and 2 patients had mixed symptoms (28.57%). Sevoflurane (83.33%), nitrous oxide (50.00%), lidocaine (50.00%), and propofol (33.33%) were frequently used for general anesthesia. The medicines that the POD-J group took most commonly were fentanyl analogs (71.43%), fentanyl (42.86%), followed by benzodiazepines (71.43%), midazolam (57.14%), and lorazepam (14.29%). In the POD-Y group, the incidence rates of adverse events, including abdominal pain (14.29%) and heart failure (14.29%), and the use of mechanical ventilation (14.29%), intensive care unit (14.29%), and recurrence rate (14.29%), were low. A total of 3 of the patients in the POD-J group died. Subsequently, we compared the clinical characteristics and outcomes between the POD-J and POD-E groups and found that the incidence of post-operative delirium following neurological surgeries was higher in the POD-J group than in the POD-E group (28.57% vs. 0.00%, *P* = 0.016). Additionally, sevoflurane (83.33% vs. 16.67%, *P* = 0.026) and nitrous oxide (50.00% vs. 0.00%, *P* = 0.044) were more frequently used for anesthesia in the POD-J group than in the POD-E group. Furthermore, fentanyl analogs (71.43% vs. 22.22%, *P* = 0.032) were more commonly administered to the POD-J group than to the POD-E group. Notably, patients in the POD-J group have a higher mortality rate. Furthermore, there is no significant statistical difference in mortality rates between the POD-Y and POD-J groups. Figure 2A effectively illustrates the data from Table 2, while Figure 3B depicts a volcano plot that highlights the differences and significance in clinical characteristics and outcomes between the POD-J group and the POD-E group.

### 3.4 Clinical features and prognosis of patients with different subtypes of post-operative delirium

According to the Diagnostic and Statistical Manual of Mental Disorders 5th edition (DSM-5), delirium is categorized into three subtypes: hyperactive delirium, hypoactive delirium and mixed delirium. The clinical manifestations of these subtypes range from a nearly unconscious state to a highly agitated state (17). In this study, we described the clinical characteristics and prognosis associated with each subtype, drawing insights from the analysis of case reports. The data are presented in Table 3.

**Table 3:** Clinical characteristics and outcomes of patients with different subtypes of post-operative delirium.

**Clinical characteristics**	**Hypoactive**	**Hyperactive**	**Mixed**	***p*-value^a^**
Number of patients	38	63	15	
Age (all)	60.58 ± 20.81	53.97 ± 23.01	52.67 ± 21.75	0.626
Age (female)	63.06 ± 19.56	51.93 ± 21.40	52.78 ± 22.40	0.224
Age (male)	58.35 ± 21.64	54.65 ± 24.18	52.50 ± 19.20	0.807
Male	20/38 (52.63%)	35/63 (55.56%)	6/15 (40.00%)	0.555
**Reasons for surgery**				
Skeletal disorders	5/38 (13.16%)	14/63 (22.22%)	3/15 (20.00%)	0.444
Cardiovascular diseases	7/38 (18.42%)	15/63 (23.81%)	0/15 (0.00%)	**0.027**
Gastrointestinal diseases	9/38 (23.68%)	8/63 (12.70%)	5/15 (33.33%)	0.133
Malignant neoplasm	4/38 (10.53%)	8/63 (12.70%)	0/15 (0.00%)	0.163
Respiratory diseases	5/38 (13.16%)	2/63 (3.17%)	0/15 (0.00%)	0.120
Ophthalmic diseases	3/38(7.89%)	0/63 (0.00%)	0/15 (0.00%)	0.077
Reproductive system diseases	2/38 (5.26%)	1/63 (1.59%)	3/15 (20.00%)	**0.043**
Neurological disorders	0/38 (0.00%)	5/63 (7.94%)	0/15 (0.00%)	**0.023**
Urological disorders	0/38 (0.00%)	2/63 (3.17%)	1/15 (6.67%)	0.234
Others	3/38 (7.89%)	8/63 (12.70%)	3/15 (20.00%)	0.476
**Anesthesia**				
Regional	2/26 (7.69%)	3/56 (5.36%)	3/12 (25.00%)	0.222
General	24/26 (92.31%)	53/56 (94.64%)	9/12 (75.00%)	0.222
**Preexisting cognitive dysfunction**	6/34 (17.65%)	10/61 (16.39%)	0/14 (0.00%)	0.163
**Delirium history**	0/33 (0.00%)	1/61 (1.64%)	1/14 (7.41%)	0.335
**Disease history**				
Smoke	1/33 (3.03%)	6/57 (10.53%)	2/15 (13.33%)	0.515
Alcohol	2/33 (6.06%)	6/57 (21.05%)	1/15 (6.67%)	0.811
Drugs (cocaine etc.)	0/33 (0.00%)	7/57 (12.28%)	0/15 (0.00%)	**0.020**
Common chronic disease	16/33 (48.48%)	22/57 (38.60%)	3/15 (20.00%)	0.164
Dyslipidemia	5/33 (15.15%)	3/57 (5.26%)	0/15 (0.00%)	0.131
Diabetes	7/33 (21.21%)	6/57 (21.05%)	1/15 (6.67%)	0.307
Hypertension	8/33 (24.24%)	14/57 (24.56%)	2/15 (13.33%)	0.657
Coronary heart disease	2/33 (6.06%)	5/57 (8.77%)	1/15 (6.67%)	0.884
Asthma	3/33 (9.09%)	2/57 (3.51%)	0/15 (0.00%)	0.424
COPD	1/33 (3.03%)	4/57 (7.02%)	0/15 (0.00%)	0.690
**Time of surgery**				
Mean ± SD min	229.80 ± 162.69	206.40 ± 128.34	148.00 ± 76.79	0.601
**Comorbidities**				
Cardiovascular	14/33 (42.42%)	21/59 (35.59%)	4/15 (26.67%)	0.567
Gastrointestinal	6/33 (18.18%)	8/59 (13.56%)	4/15 (26.67%)	0.557
Central nervous system	6/33 (18.18%)	9/59 (28.81%)	3/15 (20.00%)	0.879
Respiratory	7/33 (21.21%)	8/59 (13.56%)	0/15 (0.00%)	0.081
Hematological	1/33 (3.03%)	2/59 (3.39%)	2/15 (13.33%)	0.266
Urinary	4/33 (12.12%)	8/59 (13.56%)	1/15 (6.67%)	0.787
Mental disorders	7/33 (21.21%)	12/59 (20.34%)	3/15 (20.00%)	1
Anxiety	1/33 (3.03%)	4/59 (6.78%)	1/15 (6.67%)	0.725
Depression	3/33 (9.09%)	3/59 (5.08%)	1/15 (6.67%)	0.862
Cancer	3/33 (9.09%)	11/59 (18.64%)	2/15 (13.33%)	0.800
Else	16/33 (48.48%)	9/59 (28.81%)	2/15 (13.33%)	0.002
**Anesthetic drugs**				
Sevoflurane	3/8 (37.50%)	14/34 (41.18%)	1/7 (14.29%)	0.438
Isoflurane	3/8 (37.50%)	3/34 (8.82%)	1/7 (14.29%)	0.188
Nitrous oxide	2/8 (25.00%)	4/34 (11.76%)	2/7 (28.57%)	0.608
Propofol	5/8 (62.50%)	20/34 (58.82%)	4/7 (57.14%)	1
Ketamine	2/8 (25.00%)	2/34 (5.88%)	1/7 (14.29%)	0.160
Thiopental sodium	1/8 (12.50%)	2/34 (5.88%)	0/7 (0.00%)	0.572
Lidocaine	2/8 (25.00%)	10/34 (29.41%)	4/7 (57.14%)	0.428
Bupivacaine	1/8 (12.50%)	1/34 (2.94%)	1/7 (14.29%)	0.218
**Medicine**				
Fentanyl analogs	6/31 (19.35%)	29/58 (50.00%)	5/13 (38.46%)	**0.017**
Fentanyl	1/31 (3.23%)	16/58 (27.59%)	1/13 (7.70%)	**0.006**
Sufentanil	1/31 (3.23%)	2/58 (3.45%)	2/13 (15.39%)	0.190
Remifentanil	0/31 (0.00%)	3/58 (5.17%)	2/13 (15.39%)	0.101
Morphine	5/31 (16.13%)	11/58 (18.97%)	5/13 (38.46%)	0.267
Hydrocodone	3/31 (9.68%)	5/58 (8.62%)	0/13 (0.00%)	0.459
Succinylcholine	3/31 (9.68%)	3/58 (5.17%)	1/13 (7.70%)	0.542
Vecuronium	3/31 (9.68%)	2/58 (3.45%)	0/13 (0.00%)	0.401
Rocuronium	4/31 (12.90%)	5/58 (8.62%)	0/13 (0.00%)	0.300
Benzodiazepines	6/31 (19.35%)	33/58 (56.90%)	10/13 (76.92%)	**0.003**
Lorazepam	0/31 (0.00%)	12/58 (20.69%)	4/13 (30.77%)	**0.002**
Midazolam	6/31 (19.35%)	18/58 (31.03%)	6/13 (46.15%)	0.213
Dexmedetomidine	1/31 (3.23%)	7/58 (12.07%)	2/13 (15.38%)	0.424
Diphenhydramine	1/31 (3.23%)	2/58 (3.45%)	3/13 (23.08%)	**0.038**
Chlorpromazine	0/31 (0.00%)	5/58 (8.62%)	0/13 (0.00%)	0.245
Flupentixol	4/31 (12.90%)	23/58 (39.66%)	4/13 (30.77%)	**0.025**
Quetiapine	1/31 (3.23%)	8/58 (13.79%)	1/13 (7.70%)	0.327
Olanzapine	0/31 (0.00%)	6/58 (10.34%)	1/13 (7.70%)	0.140
NSAIDs	8/31 (25.81%)	9/58 (15.52%)	3/13 (23.08%)	0.533
Aspirin	4/31 (12.90%)	2/58 (3.45%)	1/13 (7.70%)	0.208
Atropine	3/31 (9.68%)	3/58 (5.17%)	4/13 (30.77%)	0.068
Neostigmine	5/31 (16.13%)	3/58 (5.17%)	1/13 (7.70%)	0.355
Glucocorticoids	5/31 (16.13%)	7/58 (12.07%)	3/13 (23.08%)	0.572
Furosemide	3/31 (9.68%)	2/58 (3.45%)	2/13 (15.38%)	0.172
Catecholamines	6/31 (19.35%)	4/58 (6.90%)	4/13 (30.77%)	0.055
Epinephrine	2/31 (6.45%)	2/58 (3.45%)	3/13 (23.08%)	**0.046**
Norepinephrine	3/31 (9.68%)	2/58 (3.45%)	0/13 (0.00%)	0.401
Dopamine	1/31 (3.23%)	2/58 (3.45%)	1/13 (7.70%)	0.594
Statins	1/31 (3.23%)	3/58 (5.17%)	1/13 (7.70%)	0.670
Ondansetron	0/31 (0.00%)	7/58 (12.07%)	2/13 (15.38%)	**0.043**
Antibiotics	5/31 (16.13%)	13/58 (22.41%)	5/13 (38.46%)	0.313
**Adverse events**				
Post-operative hypotension	2/30 (6.67%)	3/59 (5.08%)	1/15 (6.67%)	1
Post-operative hypertension	2/30 (6.67%)	3/59 (5.08%)	1/15 (6.67%)	1
Arrhythmia	6/30 (20.00%)	8/59 (13.56%)	4/15 (26.67%)	0.521
Anemia	2/30 (6.67%)	2/59 (3.39%)	2/15 (13.33%)	0.273
Kidney failure	2/30 (6.67%)	1/59 (1.69%)	2/15 (13.33%)	0.078
Else	21/30 (70.00%)	19/59 (32.20%)	9/15 (60.00%)	**0.002**
**ICU**	7/24 (29.17%)	21/60 (35.00%)	4/14 (28.57%)	0.823
**Mechanical ventilation**	9/24 (37.50%)	19/60 (31.67%)	4/14 (28.57%)	0.863
**Days of hospitalization**				
Mean ± SD day	32.31 ± 38.98	26.73 ± 44.88	40.00 ± 48.09	0.851
**Recurrence**	4/33 (12.12%)	9/60 (15.00%)	4/14 (28.57%)	0.430
**Follow-up**	11/38 (28.95%)	14/63 (22.22%)	3/15 (20.00%)	0.747
**Outcome**				
Death (all)	4/36 (11.11%)	4/62 (6.45%)	3/15 (20.00%)	0.390
Death (female)	1/16 (6.25%)	2/27 (7.41%)	1/9 (11.11%)	1
Death (male)	3/20 (15.00%)	2/35 (5.71%)	2/6 (33.33%)	0.128

POD, post-operative delirium; y, year; COPD, chronic obstructive pulmonary disease; SD, Standard Deviation; min, minutes; NSAIDs, non-steroidal anti-inflammatory drugs; ICU, intensive care unit. ^a^Means comparison between hypoactive group, hyperactive group and mixed group using Chi-Squared test. The bold values refer to statistical significance (*P* < 0.05).

Among 116 patients, 38 were diagnosed with hypoactive delirium, 63 with hyperactive delirium, and 15 with mixed delirium. The mean age and the proportion of males in the patient population for these groups were as follows: 60.58 ± 20.81 years and 52.63% male, 53.97 ± 23.01 years and 55.56% male, and 52.67 ± 21.75 years and 40.00% male, respectively. The predominant reasons for surgery in these cohorts were gastrointestinal diseases (Hypoactive group, 23.68%), cardiovascular diseases (Hyperactive group, 23.81%) and gastrointestinal diseases (Mixed group, 33.33%). General anesthesia was administered to the majority of patients in all three cohorts (92.31%, 94.64%, and 75.00%). A minority of patients in these groups had preexisting cognitive dysfunction (17.65%, 16.39%, and 0.00%) and a history of delirium (0.00%, 1.64%, and 7.41%). The patients in these three cohorts frequently had common chronic diseases (48.48%, 38.60%, and 20.00%), including hypertension (24.24%, 24.56%, and 13.33%), diabetes (21.21%, 21.05%, and 6.67%), dyslipidemia (15.15%, 5.26%, and 0.00%), coronary heart disease (6.06%, 8.77%, and 6.67%), asthma (9.09%, 3.51%, and 0.00%), and chronic obstructive pulmonary disease (3.03%, 7.02%, and 0.00%). The average durations of surgery in these three cohorts were 229.80 ± 162.69, 206.40 ± 128.34, and 148.00 ± 76.79, respectively. Cardiovascular diseases were the most common comorbidities in both the hypoactive and hyperactive cohorts (42.42% and 35.59%, respectively), while the mixed cohort exhibited a greater incidence of cardiovascular diseases (26.67%) and gastrointestinal diseases (26.67%). Additionally, propofol (62.50% and 58.82%) was most frequently used for anesthesia in the hypoactive cohort and hyperactive cohort, and propofol (57.14%) and lidocaine (57.14%) were most commonly used for anesthesia in the mixed cohort. Patients in these cohorts commonly used non-steroidal anti-inflammatory drugs (25.81%, 15.52%, and 23.08%) and benzodiazepines (19.35%, 56.90%, and 76.92%) and experienced a notable incidence of arrhythmia (20.00%, 13.56%, and 26.67%). Post-surgery, mechanical ventilation (37.05%, 31.67%, and 28.57%) and intensive care unit admission (29.17%, 35.00%, and 28.57%) were prevalent across all three cohorts. The average hospitalization durations were 32.31 days (SD = 38.98 days), 26.73 days (SD = 44.88 days), and 40.00 days (SD = 48.09 days). Some patients experienced recurrence (12.12%, 15.00%, and 28.57%), with 4 patients (11.11%) in the hypoactive cohort, 4 patients (6.45%) in the hyperactive cohort, and 3 patients (20.00%) in the mixed cohort dying. Statistical analyses using the chi-square test revealed significant differences in the reasons for surgery, such as cardiovascular diseases (18.42% vs. 23.81% vs. 0.00%, *P* = 0.027), reproductive system diseases (5.26% vs. 1.59% vs. 20.00%, *P* = 0.043), and neurological disorders (0.00% vs. 7.94% vs. 0.00%, *P* = 0.023). Patients with hyperactive delirium had a history of substance abuse (0.00% vs. 12.28% vs. 0.00%, *P* = 0.020), a factor absent in the other cohorts. Notably, there were significant differences in the pharmacological treatments used among these cohorts of patients throughout their entire course of delirium. Compared with those in the other cohorts, patients in the hyperactive cohort used more fentanyl analogs (19.35% vs. 50.00% vs. 38.46%, *P* = 0.017) and flupentixol (12.90% vs. 39.66% vs. 30.77%, *P* = 0.025). Patients in the mixed cohort used more benzodiazepines (19.35% vs. 56.90% vs. 76.92%, *P* = 0.003) and diphenhydramine (3.23% vs. 3.45% vs. 23.08%, *P* = 0.038) than did patients in the other cohorts. To visualize the data, heatmaps (Figure 2B) and volcano plots (Figures 3C–E) were generated based on the data in Table 3 and Supplementary material 2.

## 4 Discussion

This study mainly investigated different age groups and delirium subtypes. Concerning age, previous research has focused primarily on the clinical manifestations and efficacy of pharmacological interventions following different surgical procedures in delirium patients aged older than 65 years (4, 9, 10) and in pediatric delirium patients (23–25). However, there is a lack of exploration into the clinical characteristics and prognosis of post-operative delirium in patients younger than 65 years. Previous research has suggested that post-operative delirium patients aged 18 to 65 typically exhibit lower incidence rates and experience more favorable outcomes compared to the elderly population, which is consistent with our findings (26). However, what has not been reported previously is that post-operative delirium patients younger than 18, as well as those aged 18 to 65 with mixed-subtype delirium, exhibited similar high mortality rates as elderly patients aged 65 and above (14.29% vs. 20.45%, *P* = 1). This implies that both concealed clinical presentations and poor prognoses may coexist in these two specific patient populations. Although this finding awaits confirmation from larger-scale clinical studies, it suggests the critical need for early recognition and intervention of these low-incidence, clinically subtle but high-risk patient groups in clinical practice. Our findings also indicate that patients under the age of 65 (POD-J and POD-Y) are more likely to use fentanyl analogs, and patients in the POD-Y cohort are more inclined to use lorazepam than are those aged 65 and above. This finding suggested a potential association between fentanyl analogs and delirium in patients in the POD-Y and POD-J cohorts. Additionally, one systematic review reported that opioid medications, including fentanyl, increase the risk of delirium (27), and another randomized controlled trial conducted on ventilated patients revealed that, when used for analgesia and sedation, fentanyl resulted in a greater incidence of delirium in hospitalized patients than did morphine (28). Conversely, the administration of benzodiazepines does not increase the incidence of post-operative delirium. However, this conclusion is based on lower-quality evidence (29). Therefore, determining whether benzodiazepines can increase the risk of delirium requires clinical randomized controlled trials with larger sample sizes, as well as systematic reviews and meta-analyses based on higher-quality evidence (30). General anesthesia is considered to potentially induce short-term neurological toxicity (such as post-operative delirium) and long-term cognitive impairment in children (31, 32). Among the patients under 18 years (POD-J), sevoflurane and nitrous oxide were more commonly used for general anesthesia than were used in the POD-E group. Sevoflurane and desflurane can lead to early awakening after anesthesia, potentially contributing to the development of delirium (33), and the use of nitrous oxide may result in subacute toxic delirium (34). However, in the elderly patient population, there was no increase in the incidence of post-operative delirium in patients exposed to nitrous oxide compared to those not exposed to nitrous oxide (35). Therefore, the use of sevoflurane may be associated with the occurrence of post-operative delirium in children, but the relationship between nitrous oxide and post-operative delirium is still unclear. Previous research has emphasized the strong association between different subtypes and distinct clinical presentations and prognoses. Specifically, hyperactive delirium has shown significant correlations with factors such as age, procalcitonin levels, frailty, and overall health status (36). A survey revealed that the hypoactive subtype was the most common subtype in ICU (20). Additionally, hyperactive delirium is easier to recognize and treat, while hypoactive delirium, characterized by lethargy and confusion, is often misdiagnosed. Hypoactive delirium, due to delayed diagnosis, has the highest mortality rate and consumes more healthcare resources (36, 37). In addition, studies have also revealed the incidence and prevalence of different delirium subtypes. To further understand the pathophysiological mechanisms of post-operative delirium in these three subtypes, more clinical data and specific analyses related to these subtypes are required (4, 38–40). Our study addresses this gap by conducting a retrospective analysis of the clinical characteristics and prognosis of hyperactive, hypoactive, and mixed delirium patients. Our data revealed a significant association between hyperactive disease and a history of substance abuse. Additionally, our findings align with a study on acute delirium and non-compliance in patients with a history of substance abuse after total hip replacement surgery, suggesting that a cumulative effect of excessive alcohol use and drug abuse increases the risk of post-operative delirium and psychiatric disorders (41). This suggests that patients with a history of substance abuse may, during their hospital stay, eventually manifest hyperactive delirium as a result of the combined effects of withdrawal reactions and delirium (42). Thus, in patients with a history of substance abuse and multiple post-operative delirium risk factors (43), heightened vigilance is crucial to anticipate hyperactive delirium and implement timely intervention measures. Our study also highlights associations, such as the high usage of fentanyl and flupentixol with hyperactive delirium and the high usage of diphenhydramine, benzodiazepines, epinephrine, and ondansetron with mixed delirium. Different delirium subtypes involve distinct pathophysiological mechanisms, and various drugs potentially contribute to these mechanisms, resulting in imbalances in neurotransmitters such as acetylcholine, gamma-aminobutyric acid, and dopamine, ultimately leading to the manifestation of different subtypes in patients (44).

## 5 Limitations

Previous studies have often focused on elderly post-operative delirium patients aged over 65, with relatively less attention paid to those under 65. This younger population tends to present with a more insidious onset and a lower incidence of delirium, and is often overlooked by clinical practitioners. Therefore, we chose to collect case reports from online databases as the data source for our systematic review. However, case reports are generally considered to be of lower evidence quality and cannot provide the same level of inference as higher-tier studies such as randomized controlled trials (RCTs) or cohort studies. Additionally, there is significant heterogeneity among case reports, with variations in study design, data collection methods, diagnostic criteria, and interventions. This heterogeneity complicates data synthesis and may affect the overall consistency of the systematic review. Online case reports also tend to highlight rare or unique cases, while more common or negative outcomes may be underreported, leading to publication bias. Moreover, not all case reports adhere to standardized reporting guidelines, and some may lack critical data or details, which can affect the comprehensiveness and accuracy of the systematic review. In addition, it is challenging to control for potential confounding variables in the study, such as specific surgical techniques, the complex effects of multiple comorbidities, and perioperative management protocols, all of which may influence the measurement of delirium incidence and outcomes.

## 6 Conclusions

Our investigation has revealed noteworthy insights into post-operative delirium in different patient populations. Notably, age emerged as a pivotal factor. Compared to the POD-E group, the POD-Y group demonstrate fewer chronic medical conditions, fewer comorbidities, a lower incidence of adverse events, and lower mortality rates. Additionally, the POD-J group have higher rates of fentanyl analog use and a higher incidence of delirium following neurosurgical procedures compared to the POD-E group. Additionally, a strong association was found between a history of substance abuse and hyperactive delirium. Variations in drug use patterns were observed across different subtypes, indicating potential differences in symptomatology and clinical presentations. Importantly, post-operative delirium patients younger than 18 years (POD-J group), as well as those aged 18 to 65 with mixed-subtype delirium, exhibited similar high mortality rates as elderly patients. This underscores the need for increased attention to post-operative delirium patients under 65 and highlights the necessity of rapid identification and early intervention for these populations at risk of poor outcomes.

## Data Availability

The original contributions presented in the study are included in the article/Supplementary material, further inquiries can be directed to the corresponding author.
