# Supplementary material for: Post-operative delirium in different age groups and subtypes: a systematic review of case reports
Source: Front Neurol. 2024 Oct 10;15:1465681. doi: 10.3389/fneur.2024.1465681 (PMC11499180; doi:10.3389/fneur.2024.1465681)
Supplement: Supplementary file 1 [file Table_1.DOCX]

Supplementary Material

## Supplementary Tables

**
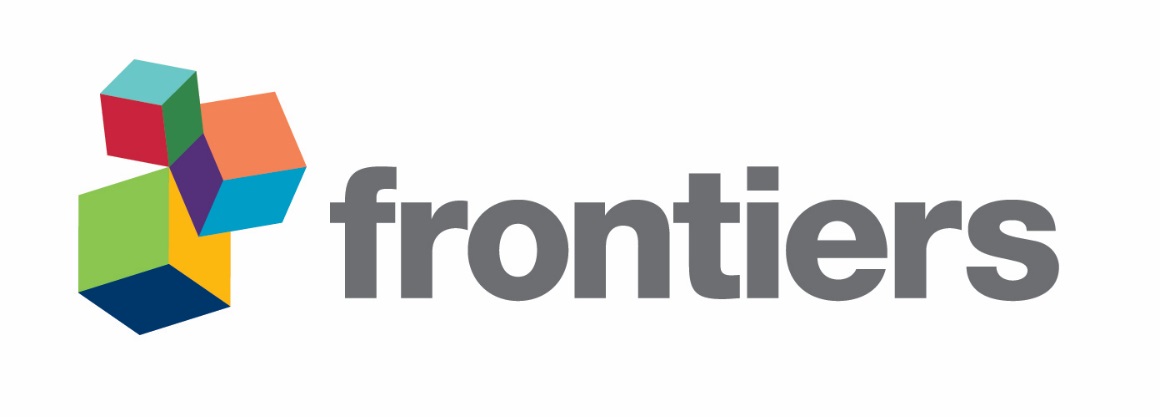
**

**Supplementary Material 1: Case report review of patients with postoperative delirium.**

| **Rank** | **Year** | **Gender** | **Age** | **Surgery** | **Disease history** | **Anesthesia** | **Time of surgery** | **Subtypes** | **Comorbidities** | **Medicine** | **Adverse Events** | **ICU** | **Mechanical ventilation** | **Recurrence** | **Outcome** | **Reference** |
| --- | --- | --- | --- | --- | --- | --- | --- | --- | --- | --- | --- | --- | --- | --- | --- | --- |
| 1 | 2023 | Female | 64 | Elective T10-S1 nerve decompression with fusion for chronic idiopathic scoliosis | Hypertension, | NA | NA | Mixed | Gastrointestinal disease, Anxiety | BZDs, Lorazepam, Midazolam, Olanzapine, Antibiotics | Urinary retention | Yes | Yes | No | Favorable | S1 |
| 2 | 2023 | Female | 42 | Craniopharyngioma resection | Else | General | NA | Hypoactive | Central nervous system disease, Cancer | Flupentixol, DA | Else | No | No | No | Favorable | S2 |
| 3 | 2023 | Male | 42 | Open reduction and internal fixation of the hip | Hypertension, Alcohol abuse | NA | NA | Hyperactive | Cardiovascular disease Gastrointestinal disease | Morphine, BZDs | Else | No | No | No | Favorable | S3 |
| 4 | 2022 | Male | 79 | Spine operations | Dyslipidemia, Diabetes, Hypertension, Asthma | General | 174mins | Hyperactive | Cardiovascular disease Respiratory disease, Urinary tract disease | Lidocaine, Morphine, Baclofen, NSAIDs | Else | No | No | Yes | Favorable | S4 |
| 5 | 2022 | Female | 69 | Aortic valve replacement | Else | General | NA | Hyperactive | Cardiovascular disease | Dexmedetomidine, Flupentixol, NE | Postoperative hypertension | Yes | Yes | No | Favorable | S5 |
| 6 | 2022 | Male | 73 | Aortic valve surgery. | Else | General | NA | Hypoactive | Cardiovascular disease Urinary tract disease | Fentanyl analogs, NSAIDs, Aspirin, NE | Abdominal pain, Arrhythmia, Hypoxemia | Yes | Yes | No | Favorable | S6 |
| 7 | 2021 | Female | 56 | Massive irrigation and thick polyethylene liner change of the knee | Diabetes | Regional | NA | Hypoactive | Mental Disorders | NA | Else | No | No | No | Favorable | S7 |
| 8 | 2021 | Female | 65 | Elective open decompression with posterior spinal fusion | Diabetes, Hypertension | General | NA | Hyperactive | Cardiovascular disease Central nervous system disease, Hematological disease, Urinary tract disease | BZDs, Midazolam, Flupentixol, Gabapentin, Metoclopramide, Glucocorticoids, Statin, Antibiotics | Postoperative hypertension, Heart failure, Stroke, Hyperglycemia, Anemia | No | No | No | Death | S8 |
| 9 | 2021 | Female | 88 | Hartmann surgery | Diabetes | General | NA | Hypoactive | Cancer | Propofol, Ketamine, Fentanyl, Rocuronium Bromide, NE, Antibiotics | Hyperglycemia, | Yes | Yes | No | Favorable | S9 |
| 10 | 2021 | Male | 29 | Appendectomy | Else | General | 120mins | Hypoactive | Gastrointestinal disease | Sevoflurane, Propofol, Sufentanyl, Rocuronium Bromide, BZDs, Midazolam, Dexmedetomidine, Atropine, Neostigmine | Else | No | No | No | Favorable | S10 |
| 11 | 2021 | Female | 72 | Elective endovascular aneurysm repair of a thoracic aortic aneurysm. | COPD, Asthma | General | NA | Hypoactive | Cardiovascular disease, respiratory disease | Furosemide | Headache and dizziness, | NA | NA | No | Favorable | S11 |
| 12 | 2021 | Female | 100 | Acetabular reconstruction | Hypertension | General | NA | Hypoactive | Cardiovascular disease | NA | Else | No | No | Yes | Favorable | S12 |
| 13 | 2021 | Female | 20 | Stereo electroencephalography | Substance abuse | General | NA | Hyperactive | Central nervous system disease | BZDs, Lorazepam, Flupentixol | NA | Yes | No | No | Favorable | S13 |
| 14 | 2021 | Male | 34 | Stereo electroencephalography | Substance abuse | General | NA | Hyperactive | Central nervous system disease | BZDs, Lorazepam, Quetiapine | NA | No | No | NA | Favorable | S13 |
| 15 | 2021 | Male | 57 | Remove cerebellar lesions | Else | General | NA | Hyperactive | Else | Propofol, Dexmedetomidine, Chlorpromazine, Quetiapine | Else | Yes | Yes | NA | Favorable | S14 |
| 16 | 2021 | Female | 68 | Total arch repair with the frozen elephant trunk technique and left subclavian artery reimplantation | Hypertension | NA | 284mins | Hypoactive | Cardiovascular disease Central nervous system disease, Hematological disease | NA | Arrhythmia, Lung infection, Kidney failure | No | No | No | Favorable | S15 |
| 17 | 2021 | Male | 22 | Superficial temporal artery (STA)–middle cerebral artery (MCA) bypass | Else | General | NA | Hypoactive | Else | NA | Else | No | No | No | Favorable | S16 |
| 18 | 2020 | Male | 37 | Emergency surgery to replace the ascending aorta with a tube graft | Substance abuse | General | NA | Hyperactive | Cardiovascular disease | BZDs, Lorazepam, Midazolam, Flupentixol, Antibiotics | Diarrhea | Yes | No | No | Favorable | S17 |
| 19 | 2020 | Male | 63 | Posterior decompression and morselized interbody bone graft with pedicle screw fixation was performed | Else | General | NA | Hyperactive | Else | BZDs, Lorazepam, Flupentixol, NSAIDs, Aspirin, PPI, Statin, Antibiotics | Else | Yes | No | No | Favorable | S18 |
| 20 | 2020 | Male | 28 | Serial endoscopic ablations | Else | General | NA | Hyperactive | Gastrointestinal disease | Sevoflurane, Propofol, Fentanyl analogs, Dexmedetomidine, PPI | Else | No | No | Yes | Favorable | S19 |
| 21 | 2020 | Male | 82 | Valvular replacement | Hypertension | General | NA | Hypoactive | Cardiovascular disease | NSAIDs, Aspirin | Postoperative hypotension, Cardiopulmonary arrest, Acute myocardial infarction, | Yes | Yes | Yes | Death | S20 |
| 22 | 2019 | Female | 39 | A combination of hysteroscopy, endometrial polypectomy and fractional curettage | Else | General | NA | Mixed | Hematological disease, | Propofol, Remifentanil, BZDs, Midazolam, Dexmedetomidine, Atropine, Glucocorticoids, Furosemide, DA | Pulmonary edema | Yes | Yes | No | Favorable | S21 |
| 23 | 2019 | Female | 58 | Total hip arthroplasty | Else | General | 120mins | Hyperactive | Central nervous system disease, Anxiety, Depression | Sevoflurane, Propofol, Ropivacaine, Fentanyl analogs, Baclofen, Rocuronium Bromide, BZDs, Chlorpromazine | Else | Yes | Yes | Yes | Favorable | S22 |
| 24 | 2019 | Female | 50 | Elective C3 to C4 anterior discectomy and fusion (ACDF) with donor bone and plating | Else | General | 101mins | Hyperactive | Central nervous system disease | Isoflurane, Propofol, Lidocaine, Fentanyl analogs, Oxymorphone, Rocuronium Bromide, BZDs, Midazolam, Neostigmine, Glucocorticoids, Ondansetron, Antibiotics | Nausea and vomiting | No | No | No | Favorable | S23 |
| 25 | 2019 | Female | 69 | Single lung transplant | Else | NA | NA | Hypoactive | Respiratory disease, Mental Disorders | NA | NA | Yes | Yes | No | Favorable | S24 |
| 26 | 2019 | Female | 57 | Bilateral lung transplant | Else | NA | NA | Hypoactive | Respiratory disease, Mental Disorders | NA | NA | NA | NA | No | Favorable | S24 |
| 27 | 2019 | Male | 69 | Bilateral lung transplant | Else | NA | NA | Hypoactive | Respiratory disease, Mental Disorders | NA | NA | NA | NA | No | Favorable | S24 |
| 28 | 2019 | Male | 55 | Liver transplant | Dyslipidemia, Asthma | General | NA | Hypoactive | Gastrointestinal disease, Central nervous system disease, respiratory disease | Glucocorticoids | Else | NA | NA | No | Favorable | S25 |
| 29 | 2018 | Female | 49 | Nephrectomy | Else | General | 200mins | Hypoactive | Else | Sevoflurane, Propofol, Tramadol, Rocuronium Bromide, BZDs, Midazolam, NSAIDs, Atropine, Neostigmine, Metoclopramide, Ranitidine | Else | No | No | No | Favorable | S26 |
| 30 | 2018 | Female | 45 | Partial laryngectomy. | Smoker | General | 210mins | Hyperactive | Cancer | Sevoflurane, Sufentanyl, Oxycodone, Zuclopenthixol, Antibiotics | Cough | Yes | No | No | Favorable | S27 |
| 31 | 2018 | Male | 4 | Myringotomy surgery. | Else | General | 37mins | Hyperactive | Else | Sevoflurane, Fentanyl analogs, Ondansetron | Else | No | No | No | Favorable | S28 |
| 32 | 2018 | Female | 41 | Coronary-artery bypass graft. | Else | General | 253mins | Hyperactive | Else | Propofol, Remifentanil, Morphine, Atracurium, BZDs, Midazolam, Dexmedetomidine, NSAIDs, Diclofec | Else | Yes | Yes | No | Favorable | S29 |
| 33 | 2018 | Male | 64 | Combined pancreas and kidney transplantation. | NA | NA | NA | Hypoactive | NA  Else | NA | Else | NA | NA | NA | Favorable | S30 |
| 34 | 2017 | Male | 83 | Coronary artery bypass. | Diabetes, Hypertension, Coronary heart disease, | General | NA | Hypoactive | Cardiovascular disease Urinary tract disease | Lidocaine, Oxycodone, Quetiapine, NSAIDs | Postoperative hypertension, Hypoxemia, Anemia | No | Yes | Yes | Favorable | S31 |
| 35 | 2017 | Male | 33 | A 2-level anterior cervical discectomy and fusion | Alcohol abuse | General | 240mins | Mixed | Central nervous system disease, Mental Disorders | Propofol, Ketamine, Lidocaine, Sufentanyl, Remifentanil, Oxymorphone, Succinylcholine, BZDs, Midazolam, Dexmedetomidine, NSAIDs, Glucocorticoids, Ondansetron | Postoperative hypertension, Arrhythmia | No | No | No | Favorable | S32 |
| 36 | 2017 | Female | 40 | Open reduction and internal fixation of her right ankle | Smoker, Alcohol abuse, Substance abuse | General | 240mins | Hyperactive | Mental Disorders | Sevoflurane, Propofol, Lidocaine, Fentanyl analogs, Oxymorphone, Oxycodone, Succinylcholine, Rocuronium Bromide, BZDs, Midazolam, Dexmedetomidine, Quetiapine, NSAIDs, Ondansetron | Else | No | No | No | Favorable | S32 |
| 37 | 2017 | Male | 49 | A single-level posterior lumbar interbody fusion | Else | General | 120mins | Hyperactive | Urinary tract disease, Mental Disorders | Propofol, Lidocaine, Fentanyl, Rocuronium Bromide, BZDs, Midazolam, Dexmedetomidine, NSAIDs, Glucocorticoids, Ondansetron | Else | No | No | No | Favorable | S32 |
| 38 | 2017 | Female | 82 | Resection of craniopharyngioma | Diabetes | General | NA | Hypoactive | Cardiovascular disease | Glucocorticoids | Arrhythmia, Cardiopulmonary arrest, | No | No | No | Death | S33 |
| 39 | 2017 | Female | 59 | Elective reparative surgery to treat rectal prolapse and enterocele | Else | NA | NA | Hypoactive | Gastrointestinal disease | NA | Acute gastric perforation, Respiratory failure, Shock | Yes | Yes | No | Favorable | S34 |
| 40 | 2016 | Female | 82 | Left colectomy with a double barrel colostomy | Hypertension | NA | NA | Hypoactive | Cardiovascular disease Urinary tract disease | Flupentixol, NE | Else | Yes | No | No | Favorable | S35 |
| 41 | 2016 | Female | 24 | Orthognathic surgery | Else | General | 420mins | Hyperactive | Hematological disease | Isoflurane, Propofol, Remifentanil, Rocuronium Bromide, BZDs, Diazepam, Midazolam, Mirtazapine, Glucocorticoids, Antibiotics | Else | No | No | No | Favorable | S36 |
| 42 | 2016 | Male | 70 | Percutaneous nephrolithotomy | NA | General | 115mins | Hyperactive | Urinary tract disease | BZDs, Midazolam, Diclofec, Neostigmine, E, NE, DA, Ondansetron, Antibiotics | Hypoxemia, Shock | Yes | Yes | No | Favorable | S37 |
| 43 | 2016 | Male | 59 | Orthotopic liver transplant. | Else | General | NA | Hypoactive | Gastrointestinal disease, Anxiety, Depression | Mirtazapine, NSAIDs, Aspirin, Glucocorticoids, Furosemide | Else | NA | NA | No | Favorable | S38 |
| 44 | 2016 | Female | 12 | Removal of a small abdominal wall nevus | Else | General | NA | Mixed | Else | Sevoflurane, Nitrous oxide, Lidocaine, Bupivacaine, Morphine, BZDs, Midazolam, E, Ondansetron | Else | No | No | No | Favorable | S39 |
| 45 | 2016 | Male | 49 | 2-vessel off-pump coronary artery bypass graft. | Coronary heart disease | General | NA | Hyperactive | Cardiovascular disease respiratory disease | Propofol, Ketamine, Fentanyl, Oxymorphone, Oxycodone, BZDs, Midazolam, Flupentixol, Gabapentin, Quetiapine, Olanzapine, NSAIDs, Antibiotics | Postoperative hypotension, Arrhythmia | Yes | Yes | No | Favorable | S40 |
| 46 | 2015 | Female | 77 | Coronary artery bypass grafting | COPD, Coronary heart disease | General | NA | Hyperactive | Cardiovascular disease | NA | Else | No | No | No | Favorable | S41 |
| 47 | 2015 | Female | 56 | Anterior tibial artery bypass | Diabetes, Hypertension | General | 340mins | Hyperactive | Urinary tract disease | Sevoflurane, Propofol, Fentanyl, Oxymorphone, Oxycodone, BZDs, Midazolam, Diphenhydramine, Promethazine, Flupentixol, Gabapentin, Quetiapine | Urinary retention | Yes | No | Yes | Favorable | S42 |
| 48 | 2015 | Female | NA | Coiling of an anterior communicating artery | Diabetes, Hypertension, COPD | General | 123.5mins | Hyperactive | Cardiovascular disease Central nervous system disease, respiratory disease | NA | Respiratory failure | No | Yes | No | Favorable | S43 |
| 49 | 2015 | Female | 52 | Parathyroidectomy and thyroidectomy. | Dyslipidemia, Hypertension | General | NA | Hyperactive | Cardiovascular disease Mental Disorders, Cancer | Sevoflurane, Remifentanil, Flupentixol, Olanzapine, Statin | Else | No | Yes | No | Favorable | S44 |
| 50 | 2014 | Male | 84 | A bowel obstruction | Else | NA | A few hours | Hyperactive | Gastrointestinal disease | NA | Else | No | No | No | Favorable | S45 |
| 51 | 2014 | Female | 64 | Liver transplant | NA | General | NA | Hypoactive | NA | NA | Else | NA | NA | No | Favorable | S46 |
| 52 | 2014 | Male | 61 | Orchiectomy | Diabetes, | NA | NA | Hypoactive | Cardiovascular disease | Glucocorticoids, Antibiotics | Abdominal pain, Postoperative hypertension, Arrhythmia | No | No | No | Favorable | S47 |
| 53 | 2013 | Female | 76 | Surgery for removal of a polyp of the colon. | Else | NA | NA | Mixed | Cardiovascular disease Gastrointestinal disease, Hematological disease, Depression | Quetiapine, NSAIDs, Aspirin, Antibiotics | Postoperative hypotension, Arrhythmia, Anemia | Yes | No | Yes | Favorable | S48 |
| 54 | 2013 | Female | 72 | Exploratory laparotomy with lysis of adhesions and ileal resection | Else | General | NA | Hypoactive | Gastrointestinal disease, Central nervous system disease, Mental Disorders | Morphine, Oxymorphone, Oxycodone, NSAIDs | Arrhythmia | No | No | No | Favorable | S49 |
| 55 | 2013 | Female | 40 | Laparoscopic surgery | Else | General | NA | Hyperactive | Urinary tract disease, Mental Disorders | NA | Else | No | No | No | Favorable | S50 |
| 56 | 2013 | Male | 78 | Hemipelvectomy | Dyslipidemia, Hypertension, Asthma, | General | NA | Hypoactive | Cardiovascular disease Respiratory disease, Urinary tract disease, Cancer | NA | Arrhythmia, Kidney failure | No | No | No | Death | S51 |
| 57 | 2012 | Male | 67 | Right upper lobectomy | Alcohol abuse | General | NA | Hypoactive |  | Bupivacaine, Morphine, BZDs, Midazolam, NSAIDs, E, Antibiotics | Nausea and vomiting, Lung infection, Respiratory failure, | Yes | Yes | No | Death | S52 |
| 58 | 2011 | Male | 55 | Cervical open-door laminoplasty | Else | NA | 200mins | Mixed | Central nervous system disease | NA | Headache and dizziness, Cerebrospinal fluid leak, Hematoma in neck | No | No | No | Favorable | S53 |
| 59 | 2011 | Male | 7m | Bidirectional Glenn procedure and tricuspid valve annuloplasty. | Else | General | NA | Hyperactive | Else | Fentanyl analogs, Morphine, BZDs, Lorazepam, Olanzapine, NSAIDs | Heart failure | Yes | Yes | Yes | Death | S54 |
| 60 | 2011 | Male | 78 | Right total knee | Dyslipidemia, Diabetes, Hypertension | NA | NA | Hypoactive | Cardiovascular disease Gastrointestinal disease, Depression | Morphine, Oxycodone, Baclofen, Diphenhydramine, Flupentixol, Gabapentin, NSAIDs, Aspirin, PPI, Ranitidine, Furosemide, Statin, Antibiotics | Else | No | No | Yes | Favorable | S55 |
| 61 | 2011 | Male | 77 | Combined TurP and cyst lithotripsy. | Dyslipidemia, Hypertension, Coronary heart disease | Regional | NA | Hyperactive | Cardiovascular disease Urinary tract disease | Propofol, Fentanyl, BZDs, Chlorpromazine, Flupentixol, Quetiapine, Olanzapine, Ondansetron, Antibiotics | Else | Yes | Yes | No | Favorable | S56 |
| 62 | 2011 | Male | 85 | Aortic valve replacement and coronary artery bypass graft. | Coronary heart disease | General | NA | Hyperactive | Cardiovascular disease Urinary tract disease | Dexmedetomidine | Kidney failure | Yes | Yes | No | Death | S57 |
| 63 | 2010 | Female | 81 | Laparoscopic colon surgery | Hypertension, Smoker | General | 180mins | Mixed | Cardiovascular disease Gastrointestinal disease | Desflurane, Propofol, Sufentanyl, Morphine, Atracurium, BZDs, Bromazepam, Midazolam, NSAIDs, Atropine, Neostigmine, Statin, Antibiotics | Diarrhea, Heart failure, Respiratory failure, Kidney failure | No | Yes | Yes | Favorable | S58 |
| 64 | 2010 | Male | 85 | Left lobectomy of the liver and lymph node dissection | Else | NA | NA | Hyperactive | Cancer | Flupentixol, Quetiapine, Olanzapine | Urinary retention | No | No | No | Favorable | S59 |
| 65 | 2010 | Male | 46 | Coronary artery bypass graft surgery. | NA | General | NA | Hyperactive | NA | Propofol, Succinylcholine, BZDs, Lorazepam, Midazolam, Chlorpromazine, Flupentixol, Olanzapine, Atropine | Arrhythmia, Shivering | Yes | No | No | Favorable | S60 |
| 66 | 2009 | Female | 72 | Bipolar hip replacement | Hypertension, COPD | Regional | NA | Hyperactive | Cardiovascular disease Central nervous system disease, Respiratory disease, Anxiety | Propofol, Oxymorphone, BZDs, Diazepam, Antibiotics | Arrhythmia, Cardiopulmonary arrest, Lung infection | Yes | Yes | No | Death | S61 |
| 67 | 2009 | Female | 76 | Right L5 foraminotomy and L5-S1 fusion | Else | NA | NA | Hyperactive | Else | Tramadol, Glucocorticoids | NA | NA | NA | NA | NA | S62 |
| 68 | 2009 | Female | 59 | Left nephrectomy, surrenalectomy, oophorectomy, splenectomy, left colectomy and half pancreatectomy | Else | General | 341mins | Hyperactive | Cancer | Sevoflurane, Propofol, Sufentanyl, Atracurium, Flupentixol, NSAIDs | Else | Yes | No | No | Favorable | S63 |
| 69 | 2009 | Male | 95 | Cholecystectomy | Hypertension, | General | NA | Hyperactive | Cardiovascular disease Central nervous system disease, | Sevoflurane, Propofol, Lidocaine, Fentanyl | Else | No | No | Yes | Favorable | S64 |
| 70 | 2009 | Female | 74 | Gastrectomy | NA | General | NA | Hyperactive | Mental Disorders | Sevoflurane, Lidocaine, Fentanyl | Else | No | No | No | Favorable | S64 |
| 71 | 2009 | Female | 72 | Gastrectomy | NA | General | NA | Hyperactive | NA | Propofol, Fentanyl | Else | No | No | No | Favorable | S64 |
| 72 | 2009 | Female | 36 | Gynecologic surgery | NA | General | NA | Hyperactive | NA | Glucocorticoids | Else | NA | NA | NA | Favorable | S65 |
| 73 | 2008 | Male | 50 | Excision of this lobe and the overlying chest wall. | Smoker, Alcohol abuse, Substance abuse, | General | NA | Hyperactive | Gastrointestinal disease, Respiratory disease, Cancer | Bupivacaine, Fentanyl, BZDs, Lorazepam, Flupentixol | Arrhythmia | No | Yes | No | Favorable | S66 |
| 74 | 2008 | Female | 70 | Revision included removal of prior instrumentation with insertion screws from T-3 to the ilium, an L-2 pedicle subtraction osteotomy and revision L-1, L-2 and L-3 laminectomies | Dyslipidemia, Hypertension | NA | 510mins | Hypoactive | Cardiovascular disease Central nervous system disease | NA | Postoperative hypotension | No | Yes | No | Favorable | S67 |
| 75 | 2007 | Female | 78 | Exploratory laparotomy with colon resection and colostomy | Else | General | NA | Mixed | Cardiovascular disease, Cancer | Morphine, BZDs, Lorazepam, Flupentixol | Else | No | No | No | Favorable | S68 |
| 76 | 2006 | Female | 82 | Surgery for hip | NA | NA | NA | Hyperactive | NA | NA | Else | No | No | No | Favorable | S69 |
| 77 | 2006 | Male | 62 | Dental implantation | Diabetes, Hypertension | Regional | 130mins | Hyperactive | Cardiovascular disease | Lidocaine, BZDs, Midazolam, E | Postoperative hypertension | No | No | Yes | Favorable | S70 |
| 78 | 2005 | Male | 72 | Resection of hepatocellular carcinoma. | Else | NA | 540mins | Hyperactive | Gastrointestinal disease, Cancer | NA | Else | No | No | No | Favorable | S71 |
| 79 | 2005 | Male | 48 | Superficial temporal artery (STA)-MCA anastomosis | Else | General | NA | Hyperactive | Central nervous system disease | Propofol, Fentanyl | Else | No | No | No | Favorable | S72 |
| 80 | 2005 | Male | 83 | Laryngeal tumor biopsy | Hypertension | General | NA | Hyperactive | Cardiovascular disease, Cancer | Flupentixol | Else | No | No | No | Favorable | S73 |
| 81 | 2004 | Male | 68 | Craniotomy. | Else | General | NA | Mixed | Central nervous system disease | BZDs | Else | NA | NA | No | Favorable | S74 |
| 82 | 2003 | Male | 32 | Endoscopic sinus surgery | Else | General | 35mins | Hypoactive | Respiratory disease | Sevoflurane, Nitrous oxide, Propofol, Lidocaine, Fentanyl analogs, Rocuronium Bromide, BZDs, Midazolam, Neostigmine, E | NA | NA | NA | NA | Favorable | S75 |
| 83 | 2003 | Female | 47 | Facelift procedure | Substance abuse | General | 300mins | Hyperactive | Depression | Propofol, Fentanyl, BZDs, Midazolam, Flupentixol, | Else | No | No | No | Favorable | S76 |
| 84 | 2002 | Male | 87 | Surgery to resect the lung mass | Else | NA | NA | Hyperactive | Gastrointestinal disease, Respiratory disease, Mental Disorders, Cancer | Morphine, Flupentixol, NSAIDs, Aspirin, Metoclopramide, Ranitidine, | Arrhythmia | No | No | No | Favorable | S77 |
| 85 | 2002 | Male | 53 | A hip pinning procedure | Else | General | NA | Hyperactive | Gastrointestinal disease | Morphine, Oxycodone, BZDs, Lorazepam, Flupentixol, Atropine, | Else | No | No | No | Favorable | S78 |
| 86 | 2002 | Male | 78 | A hip pinning procedure | Coronary heart disease | Regional | NA | Hypoactive | Cardiovascular disease Central nervous system disease, | Morphine, BZDs, Midazolam, | Else | No | No | No | Favorable | S78 |
| 87 | 2002 | Female | 86 | Partial maxillectomy | Hypertension | General | NA | Hyperactive | Cardiovascular disease, Cancer | NA | Else | No | Yes | No | Favorable | S79 |
| 88 | 2001 | Male | 63 | Total laryngectomy | Else | General | NA | Hyperactive | Gastrointestinal disease, Anxiety, Cancer, | Morphine, BZDs, Lorazepam, Quetiapine, | Else | No | No | No | Favorable | S80 |
| 89 | 2001 | Male | 77 | Coronary artery bypass grafting | Hypertension | General | NA | Hyperactive | Cardiovascular disease | Morphine, BZDs, Midazolam, Flupentixol, | Else | Yes | Yes | No | Favorable | S81 |
| 90 | 2000 | Male | 17 | A metacarpal osteotomy for non-union of an old fracture | Else | General | 30mins | Mixed | Else | Isoflurane, Nitrous oxide, Propofol, Morphine, Pethidine, BZDs, Midazolam, | Abdominal pain | No | No | No | Favorable | S82 |
| 91 | 2000 | Female | 64 | Triple coronary artery bypass grafting surgery | Hypertension, Smoker | General | NA | Hyperactive | Cardiovascular disease | BZDs, Lorazepam, Flupentixol, Metoclopramide, Furosemide, | Arrhythmia | No | Yes | No | Favorable | S83 |
| 92 | 1999 | Female | 49 | Parathyroidectomy | Else | General | NA | Hyperactive | Depression | Nitrous oxide, Propofol, Morphine, Atracurium, BZDs, Diazepam, Diclofec, Ondansetron, Antibiotics, | Else | Yes | Yes | No | Favorable | S84 |
| 93 | 1999 | Male | 24 | Irrigation and debridement of his right fifth digit and split-thickness skin grafting | Else | General | NA | Hyperactive | Else | Sevoflurane, Nitrous oxide, Fentanyl, | Else | No | No | NA | Favorable | S85 |
| 94 | 1999 | Female | 8 | Incision and drainage of a submandibular lymph node abscess. | Else | General | NA | Hyperactive | Else | Sevoflurane, Nitrous oxide, Propofol, Lidocaine, Fentanyl, BZDs, Midazolam, | Else | No | No | No | Favorable | S85 |
| 95 | 1999 | Male | 4 | Dental rehabilitation. | Else | General | NA | Hyperactive | Else | Sevoflurane, Lidocaine, Fentanyl, BZDs, Midazolam, | Else | No | No | No | Favorable | S85 |
| 96 | 1999 | Female | 3 | Revision of a ventricular-peritoneal shunt. | Else | General | NA | Hyperactive | Else | Sevoflurane, Fentanyl, | Else | No | No | No | Favorable | S85 |
| 97 | 1999 | Male | 83 | Distal partial gastrectomy and transverse colectomy | Else | General | NA | Hypoactive | Else | Glucocorticoids, Antibiotics, | Anemia | No | No | No | Favorable | S86 |
| 98 | 1997 | Male | 66 | Left hepatic lobectomy | Diabetes | General | NA | Hyperactive | Cancer | Flupentixol, | Acute gastric perforation, Anemia, Shock | No | No | Yes | Favorable | S87 |
| 99 | 1996 | Male | 69 | Bilateral renal endarterectomy | Diabetes, Coronary heart disease, Smoker | Regional | NA | Mixed | Cardiovascular disease Urinary tract disease | Lidocaine, Fentanyl, Morphine, Pethidine, BZDs, Lorazepam, Diphenhydramine, Flupentixol, Furosemide, Antibiotics, | Arrhythmia, Chest pain, Cardiopulmonary arrest, Urticaria, kidney failure | Yes | Yes | No | Death | S88 |
| 100 | 1995 | Male | 40 | Single-lung transplantation | COPD, Smoker | General | 315mins | Hyperactive | Respiratory disease | Fentanyl analogs, Vecuronium bromide, BZDs, Midazolam, Diphenhydramine, Flupentixol, Ranitidine, Glucocorticoids, Antibiotics, | Else | No | Yes | No | Favorable | S89 |
| 101 | 1993 | Male | 64 | Extracapsular cataract extraction with posterior chamber intraocular lens implantation in the left eye. | Else | NA | NA | Hypoactive | Else | NA | Else | NA | NA | No | Favorable | S90 |
| 102 | 1993 | Female | 58 | Cryoretinopexy with segmental episcleral buckling | Else | General | NA | Hypoactive | Else | Flupentixol, | Else | NA | NA | No | Favorable | S90 |
| 103 | 1993 | Female | 26 | Cryoretinopexy. | Else | NA | NA | Hypoactive | Else | NA | Else | NA | NA | No | Favorable | S90 |
| 104 | 1993 | Male | 20 | Exploratory laparotomy | NA | General | NA | Hypoactive | NA | Isoflurane, Ketamine, Morphine, Succinylcholine, Vecuronium bromide, BZDs, Midazolam, Neostigmine, | NA | NA | NA | NA | NA | S91 |
| 105 | 1993 | Male | 19 | Appendectomy | NA | General | NA | Hypoactive | NA | Isoflurane, Nitrous oxide, Propofol, Fentanyl analogs, Succinylcholine, Vecuronium bromide, Neostigmine, | NA | NA | NA | NA | Favorable | S91 |
| 106 | 1993 | Female | 21 | Laparotomy | NA | General | NA | Hypoactive | NA | Isoflurane, Thiopental sodium, Fentanyl analogs, Succinylcholine, Vecuronium bromide, Tubocurarina, Atropine, | NA | NA | NA | NA | NA | S91 |
| 107 | 1993 | Male | 73 | Subtotal gastrectomy | Else | General | NA | Mixed | Cancer | NA | Heart failure, Lung infection, Anemia | No | No | Yes | Death | S92 |
| 108 | 1991 | Male | 29 | Right frontal sinus trephination | Asthma | General | 45mins | Hyperactive | Respiratory disease | Enflurane, Nitrous oxide, Fluothane, Ketamine, Thiopental sodium, Fentanyl, Morphine, Succinylcholine, Tubocurarina, BZDs, Diazepam, Neostigmine, Antibiotics, | Headache and dizziness | No | Yes | No | Favorable | S93 |
| 109 | 1991 | Male | 48 | Coronary revascularization | Coronary heart disease, Smoker, Alcohol abuse, Substance abuse | General | 220mins | Hyperactive | Cardiovascular disease, Anxiety | Isoflurane, Lidocaine, Fentanyl, Morphine, Vecuronium bromide, BZDs, Lorazepam, Midazolam, Flupentixol, Furosemide, DA, | Postoperative hypotension, Arrhythmia, Cardiopulmonary arrest | Yes | Yes | No | Favorable | S94 |
| 110 | 1991 | Female | 53 | Orthotopic liver transplant | Else | General | NA | Mixed | Gastrointestinal disease | Flupentixol, Glucocorticoids, Antibiotics, | Else | No | No | Yes | Death | S95 |
| 111 | 1988 | Female | 35 | Elective cesarean section | Else | Regional | 90mins | Mixed | Else | Lidocaine, Fentanyl analogs, Diphenhydramine, Flupentixol, Atropine, E, | Arrhythmia | No | No | No | Favorable | S96 |
| 112 | 1983 | Male | 58 | Surgical replacement of his mitral valve. | Else | General | NA | Hyperactive | Cardiovascular disease | Flupentixol, | Arrhythmia | Yes | No | No | Favorable | S97 |
| 113 | 1982 | Male | 51 | Coronary artery bypass grafting | Dyslipidemia, Diabetes, Smoker, Alcohol abuse | General | NA | Hypoactive | Cardiovascular disease, Depression | NA | Else | No | Yes | No | Favorable | S98 |
| 114 | 1975 | Male | 64 | A sinus resection of his hip | Else | General | 70mins | Hyperactive | Else | Thiopental sodium, Fentanyl analogs, Pethidine, Tubocurarina, BZDs, Lorazepam, Atropine, | Else | No | No | Yes | Favorable | S99 |
| 115 | 1975 | Female | 37 | Total abdominal hysterectomy. | Else | Regional | NA | Mixed | Else | Pethidine, BZDs, Lorazepam, Diphenhydramine, Atropine, E, | Nausea and vomiting | No | No | No | Favorable | S99 |
| 116 | 1970 | Female | 37 | Aortic valvotomy | Hypertension | General | 120mins | Hyperactive | Cardiovascular disease | Tubocurarina, Chlorpromazine, | Postoperative hypotension | NA | NA | No | Favorable | S100 |

Abbreviations: min, minutes; 7 m, 7 months; ICU, intensive care unit; COPD, chronic obstructive pulmonary disease; BZDs, benzodiazepines; E, epinephrine; NE, norepinephrine; DA, dopamine; PPI, proton pump inhibitor; NSAIDs, non-steroidal anti-inflammatory drugs.

**Supplementary Material 2: *P*-value of comparison between each group and every other group for *Table 2*.**

| **Clinical characteristics** | **Hypoactive** | **Hyperactive** | **Mixed** | ***p*-value^a^** | ***p*-value^b^** | ***p*-value^c^** |
| --- | --- | --- | --- | --- | --- | --- |
| Number of patients | 38 | 63 | 15 |  |  |  |
| Age (all) | 60.58±20.81 | 53.97±23.01 | 52.67±21.75 | 0.141 | 0.839 | 0.239 |
| Age (female) | 63.06±19.56 | 51.93±21.40 | 52.78±22.40 | 0.113 | 0.922 | 0.260 |
| Age (male) | 58.35±21.64 | 54.65±24.18 | 52.50±19.20 | 0.562 | 0.815 | 0.543 |
| Male | 20/38 (52.63%) | 35/63 (55.56%) | 6/15 (40.00%) | 0.775 | 0.278 | 0.407 |
| **Reasons for surgery** |  |  |  |  |  |  |
| Skeletal disorders | 5/38 (13.16%) | 14/63 (22.22%) | 3/15 (20.00%) | 0.259 | 1 | 0.841 |
| Cardiovascular diseases | 7/38 (18.42%) | 15/63 (23.81%) | 0/15 (0.00%) | 0.525 | 0.082 | 0.182 |
| Gastrointestinal diseases | 9/38 (23.68%) | 8/63 (12.70%) | 5/15 (33.33%) | 0.153 | 0.123 | 0.710 |
| Malignant neoplasm | 4/38 (10.53%) | 8/63 (12.70%) | 0/15 (0.00%) | 0.992 | 0.325 | 0.466 |
| Respiratory diseases | 5/38 (13.16%) | 2/63 (3.17%) | 0/15 (0.00%) | 0.131 | 0.650 | 0.340 |
| Ophthalmic diseases | 3/38(7.89%) | 0/63 (0.00%) | 0/15 (0.00%) | 0.097 | - | 0.360 |
| Reproductive system diseases | 2/38 (5.26%) | 1/63 (1.59%) | 3/15 (20.00%) | 0.653 | **0.021** | 0.653 |
| Neurological disorders | 0/38 (0.00%) | 5/63 (7.94%) | 0/15 (0.00%) | 0.191 | 0.333 | - |
| Urological disorders | 0/38 (0.00%) | 2/63 (3.17%) | 1/15 (6.67%) | 0.387 | 0.478 | 0.283 |
| Others | 3/38 (7.89%) | 8/63 (12.70%) | 3/15 (20.00%) | 0.674 | 0.751 | 0.440 |
| **Anesthesia** |  |  |  |  |  |  |
| Regional | 2/26 (7.69%) | 3/56 (5.36%) | 3/12 (25.00%) | 1 | 0.106 | 0.342 |
| General | 24/26 (92.31%) | 53/56 (94.64%) | 9/12 (75.00%) | 1 | 0.106 | 0.342 |
| **Preexisting cognitive dysfunction** | 6/34 (17.65%) | 10/61 (16.39%) | 0/14 (0.00%) | 0.876 | 0.233 | 0.230 |
| **Delirium history** | 0/33 (0.00%) | 1/61 (1.64%) | 1/14 (7.41%) | 0.649 | 0.341 | 0.298 |
| **Disease history** |  |  |  |  |  |  |
| Smoke | 1/33 (3.03%) | 6/57 (10.53%) | 2/15 (13.33%) | 0.384 | 1 | 0.227 |
| Alcohol | 2/33 (6.06%) | 6/57 (10.53%) | 1/15 (6.67%) | 0.739 | 1 | 0.685 |
| Drugs (cocaine etc.) | 0/33 (0.00%) | 7/57 (12.28%) | 0/15 (0.00%) | 0.091 | 0.348 | **-** |
| Comom Chronic disease | 16/33 (48.48%) | 22/57 (38.60%) | 3/15 (20.00%) | 0.360 | 0.178 | 0.061 |
| Dyslipidemia | 5/33 (15.15%) | 3/57 (5.26%) | 0/15 (0.00%) | 0.229 | 0.491 | 0.279 |
| Diabetes | 7/33 (21.21%) | 6/57 (21.05%) | 1/15 (6.67%) | 0.165 | 1 | 0.403 |
| Hypertension | 8/33 (24.24%) | 14/57 (24.56%) | 2/15 (13.33%) | 0.973 | 0.561 | 0.632 |
| Coronary heart disease | 2/33 (6.06%) | 5/57 (8.77%) | 1/15 (6.67%) | 0.957 | 1 | 0.685 |
| Asthma | 3/33 (9.09%) | 2/57 (3.51%) | 0/15 (0.00%) | 0.524 | 0.624 | 0.315 |
| COPD | 1/33 (3.03%) | 4/57 (7.02%) | 0/15 (0.00%) | 0.750 | 0.384 | 0.688 |
| Time of surgery |  |  |  |  |  |  |
| Mean ± SD min | 229.80±162.69 | 206.40±128.34 | 148.00±76.79 | 0.776 | 0.229 | 0.343 |
| **Comorbidities** |  |  |  |  |  |  |
| Cardiovascular | 14/33 (42.42%) | 21/59 (35.59%) | 4/15 (26.67%) | 0.517 | 0.514 | 0.296 |
| Gastrointestinal | 6/33 (18.18%) | 8/59 (13.56%) | 4/15 (26.67%) | 0.554 | 0.402 | 0.774 |
| Central nervous system | 6/33 (18.18%) | 9/59 (28.81%) | 3/15 (20.00%) | 0.715 | 0.958 | 1 |
| Respiratory | 7/33 (21.21%) | 8/59 (13.56%) | 0/15 (0.00%) | 0.341 | 0.296 | 0.137 |
| Hematological | 1/33 (3.03%) | 2/59 (3.39%) | 2/15 (13.33%) | 1 | 0.181 | 0.227 |
| Urinary | 4/33 (12.12%) | 8/59 (13.56%) | 1/15 (6.67%) | 1 | 0.774 | 0.949 |
| Mental Disorders | 7/33 (21.21%) | 12/59 (20.34%) | 3/15 (20.00%) | 0.921 | 1 | 1 |
| Anxiety | 1/33 (3.03%) | 4/59 (6.78%) | 1/15 (6.67%) | 0.778 | 1 | 0.532 |
| Depression | 3/33 (9.09%) | 3/59 (5.08%) | 1/15 (6.67%) | 0.759 | 0.604 | 1 |
| Cancer | 3/33 (9.09%) | 11/59 (18.64%) | 2/15 (13.33%) | 0.357 | 0.918 | 1 |
| Else | 16/33 (48.48%) | 9/59 (28.81%) | 2/15 (13.33%) | **<0.001** | 1 | **0.013** |
| **Anesthetic drugs** |  |  |  |  |  |  |
| Sevoflurane | 3/8 (37.50%) | 14/34 (41.18%) | 1/7 (14.29%) | 1 | 0.436 | 0.668 |
| Isoflurane | 3/8 (37.50%) | 3/34 (8.82%) | 1/7 (14.29%) | 0.128 | 0.542 | 0.668 |
| Nitrous oxide | 2/8 (25.00%) | 4/34 (11.76%) | 2/7 (28.57%) | 0.688 | 0.576 | 1 |
| Propofol | 5/8 (62.50%) | 20/34 (58.82%) | 4/7 (57.14%) | 1 | 1 | 1 |
| Ketamine | 2/8 (25.00%) | 2/34 (5.88%) | 1/7 (14.29%) | 0.158 | 0.439 | 1 |
| Thiopental sodium | 1/8 (12.50%) | 2/34 (5.88%) | 0/7 (0.00%) | 0.479 | 0.684 | 0.533 |
| Lidocaine | 2/8 (25.00%) | 10/34 (29.41%) | 4/7 (57.14%) | 1 | 0.331 | 0.460 |
| Bupivacaine | 1/8 (12.50%) | 1/34 (2.94%) | 1/7 (14.29%) | 0.348 | 0.316 | 0.533 |
| **Medicine** |  |  |  |  |  |  |
| Fentanyl analogs | 6/31 (19.35%) | 29/58 (50.00%) | 5/13 (38.46%) | **0.005** | 0.452 | 0.340 |
| Fentanyl | 1/31 (3.23%) | 16/58 (27.59%) | 1/13 (7.70%) | **0.005** | 0.246 | 0.508 |
| Sufentanyl | 1/31 (3.23%) | 2/58 (3.45%) | 2/13 (15.39%) | 1 | 0.151 | 0.204 |
| Remifentanil | 0/31 (0.00%) | 3/58 (5.17%) | 2/13 (15.39%) | 0.502 | 0.224 | 0.082 |
| Morphine | 5/31 (16.13%) | 11/58 (18.97%) | 5/13 (38.46%) | 0.740 | 0.249 | 0.223 |
| Hydrocodone | 3/31 (9.68%) | 5/58 (8.62%) | 0/13 (0.00%) | 1 | 0.352 | 0.339 |
| Succinylcholine | 3/31 (9.68%) | 3/58 (5.17%) | 1/13 (7.70%) | 0.716 | 0.563 | 1 |
| Vecuronium | 3/31 (9.68%) | 2/58 (3.45%) | 0/13 (0.00%) | 0.464 | 0.665 | 0.339 |
| Rocuronium | 4/31 (12.90%) | 5/58 (8.62%) | 0/13 (0.00%) | 0.788 | 0.352 | 0.433 |
| Benzodiazepines | 6/31 (19.35%) | 33/58 (56.90%) | 10/13 (76.92%) | **<0.001** | 0.182 | **0.001** |
| Lorazepam | 0/31 (0.00%) | 12/58 (20.69%) | 4/13 (30.77%) | **0.017** | 0.675 | **0.008** |
| Midazolam | 6/31 (19.35%) | 18/58 (31.03%) | 6/13 (46.15%) | 0.237 | 0.473 | 0.147 |
| Dexmedetomidine | 1/31 (3.23%) | 7/58 (12.07%) | 2/13 (15.38%) | 0.317 | 1 | 0.204 |
| Diphenhydramine | 1/31 (3.23%) | 2/58 (3.45%) | 3/13 (23.08%) | 1 | **0.040** | 0.130 |
| Chlorpromazine | 0/31 (0.00%) | 5/58 (8.62%) | 0/13 (0.00%) | 0.230 | 0.352 | - |
| Flupentixol | 4/31 (12.90%) | 23/58 (39.66%) | 4/13 (30.77%) | **0.009** | 0.779 | 0.330 |
| Quetiapine | 1/31 (3.23%) | 8/58 (13.79%) | 1/13 (7.70%) | 0.228 | 0.892 | 0.508 |
| Olanzapine | 0/31 (0.00%) | 6/58 (10.34%) | 1/13 (7.70%) | 0.158 | 1 | 0.295 |
| NSAIDs | 8/31 (25.81%) | 9/58 (15.52%) | 3/13 (23.08%) | 0.239 | 0.804 | 1 |
| Aspirin | 4/31 (12.90%) | 2/58 (3.45%) | 1/13 (7.70%) | 0.211 | 0.460 | 1 |
| Atropine | 3/31 (9.68%) | 3/58 (5.17%) | 4/13 (30.77%) | 0.716 | **0.022** | 0.196 |
| Neostigmine | 5/31 (16.13%) | 3/58 (5.17%) | 1/13 (7.70%) | 0.183 | 0.563 | 0.793 |
| Glucocorticoids | 5/31 (16.13%) | 7/58 (12.07%) | 3/13 (23.08%) | 0.835 | 0.555 | 0.907 |
| Furosemide | 3/31 (9.68%) | 2/58 (3.45%) | 2/13 (15.38%) | 0.464 | 0.151 | 0.981 |
| Catecholamines | 6/31 (19.35%) | 4/58 (6.90%) | 4/13 (30.77%) | 0.155 | **0.048** | 0.667 |
| Epinephrine | 2/31 (6.45%) | 2/58 (3.45%) | 3/13 (23.08%) | 0.909 | **0.040** | 0.287 |
| Norepinephrine | 3/31 (9.68%) | 2/58 (3.45%) | 0/13 (0.00%) | 0.464 | 0.665 | 0.339 |
| Dopamine | 1/31 (3.23%) | 2/58 (3.45%) | 1/13 (7.70%) | 1 | 0.460 | 0.508 |
| Statins | 1/31 (3.23%) | 3/58 (5.17%) | 1/13 (7.70%) | 1 | 0.563 | 0.508 |
| Ondansetron | 0/31 (0.00%) | 7/58 (12.07%) | 2/13 (15.38%) | 0.109 | 1 | 0.082 |
| Antibiotics | 5/31 (16.13%) | 13/58 (22.41%) | 5/13 (38.46%) | 0.482 | 0.396 | 0.223 |
| **Adverse Events** |  |  |  |  |  |  |
| Postoperative hypotension | 2/30 (6.67%) | 3/59 (5.08%) | 1/15 (6.67%) | 1 | 0.604 | 1 |
| Postoperative hypertension | 2/30 (6.67%) | 3/59 (5.08%) | 1/15 (6.67%) | 1 | 0.604 | 1 |
| Arrhythmia | 6/30 (20.00%) | 8/59 (13.56%) | 4/15 (26.67%) | 0.631 | 0.402 | 0.899 |
| Anemia | 2/30 (6.67%) | 2/59 (3.39%) | 2/15 (13.33%) | 0.870 | 0.181 | 0.853 |
| Kidney failure | 2/30 (6.67%) | 1/59 (1.69%) | 2/15 (13.33%) | 0.544 | 0.103 | 0.853 |
| Else | 21/30 (70.00%) | 19/59 (32.20%) | 9/15 (60.00%) | **<0.001** | **0.047** | 0.502 |
| **ICU** | 7/24 (29.17%) | 21/60 (35.00%) | 4/14 (28.57%) | 0.608 | 0.885 | 1 |
| **Mechanical ventilation** | 9/24 (37.50%) | 19/60 (31.67%) | 4/14 (28.57%) | 0.608 | 1 | 0.837 |
| **Discharge time after surgery** |  |  |  |  |  |  |
| Mean ± SD day | 32.31±38.98 | 26.73±44.88 | 40.00±48.09 | 0.684 | 0.692 | 0.820 |
| **Recurrence** | 4/33 (12.212%) | 9/60 (15.00%) | 4/14 (28.57%) | 0.944 | 0.417 | 0.343 |
| **Follow-up** | 11/38(28.95%) | 14/63 (22.22%) | 3/15(20.00%) | 0.448 | 1 | 0.749 |
| **Outcome** |  |  |  |  |  |  |
| Death (all) | 4/36 (11.11%) | 4/62 (6.45%) | 3/15 (20.00%) | 0.668 | 0.255 | 0.694 |
| Death (female) | 1/16 (6.25%) | 2/27 (7.41%) | 1/9 (11.11%) | 1 | 0.590 | 0.600 |
| Death (male) | 3/20 (15.00%) | 2/35 (5.71%) | 2/6 (33.33%) | 0.506 | 0.095 | 0.683 |

^a^Means comparison between hypoactive group and hyperactive group using Chi-Squared test.

^b^Means comparison between hyperactive group and mixed group using Chi-Squared test.

^c^Means comparison between hypoactive group and mixed group using Chi-Squared test.

**Supplementary Material 3: The results of the quality assessment using Joanna Briggs Institute Critical Appraisal tools for Case Reports.**

| **Reference** | **Question 1** | **Question 2** | **Question 3** | **Question 4** | **Question 5** | **Question 6** | **Question 7** | **Question 8** | **Total rank** |
| --- | --- | --- | --- | --- | --- | --- | --- | --- | --- |
| S1 | Yes | Yes | Yes | Yes | Yes | Yes | Yes | Yes | 8 |
| S2 | Yes | Yes | Yes | No | Yes | Yes | Yes | Yes | 7 |
| S3 | Yes | Yes | Yes | Yes | Yes | Yes | Yes | Yes | 8 |
| S4 | Yes | Yes | Yes | Yes | Yes | Yes | Yes | Yes | 8 |
| S5 | Yes | Yes | Yes | Yes | Yes | Yes | Yes | Yes | 8 |
| S6 | Yes | Yes | Yes | No | Yes | Yes | Yes | Yes | 7 |
| S7 | Yes | Yes | Yes | No | No | Yes | Yes | Yes | 6 |
| S8 | Yes | Yes | Yes | Yes | Yes | Yes | Yes | Yes | 8 |
| S9 | Yes | Yes | Yes | No | Yes | Yes | Yes | Yes | 7 |
| S10 | Yes | Yes | Yes | Yes | Yes | Yes | Yes | Yes | 8 |
| S11 | Yes | Yes | Yes | Yes | No | Yes | Yes | Yes | 7 |
| S12 | Yes | Yes | Yes | No | No | Yes | Yes | Yes | 6 |
| S13 | Yes | Yes | Yes | Yes | Yes | Yes | No | Yes | 7 |
| S14 | Yes | Yes | Yes | Yes | Yes | Yes | Yes | Yes | 8 |
| S15 | Yes | Yes | Yes | No | No | Yes | Yes | Yes | 6 |
| S16 | Yes | Yes | Yes | No | Yes | Yes | Yes | Yes | 7 |
| S17 | Yes | Yes | Yes | Yes | Yes | Yes | Yes | Yes | 8 |
| S18 | Yes | Yes | Yes | Yes | Yes | Yes | Yes | Yes | 8 |
| S19 | Yes | Yes | Yes | Yes | Yes | Yes | Yes | Yes | 8 |
| S20 | Yes | Yes | Yes | No | Yes | Yes | Yes | Yes | 7 |
| S21 | Yes | Yes | Yes | Yes | Yes | Yes | Yes | Yes | 8 |
| S22 | Yes | Yes | Yes | Yes | Yes | Yes | Yes | Yes | 8 |
| S23 | Yes | Yes | Yes | Yes | Yes | Yes | Yes | Yes | 8 |
| S24 | Yes | Yes | Yes | No | No | Yes | No | Yes | 5 |
| S25 | Yes | Yes | Yes | Yes | No | Yes | Yes | Yes | 7 |
| S26 | Yes | Yes | Yes | Yes | Yes | Yes | Yes | Yes | 8 |
| S27 | Yes | Yes | Yes | Yes | Yes | Yes | Yes | Yes | 8 |
| S28 | Yes | Yes | Yes | Yes | Yes | Yes | Yes | Yes | 8 |
| S29 | Yes | Yes | Yes | Yes | Yes | Yes | Yes | Yes | 8 |
| S30 | Yes | No | Yes | No | No | No | No | Yes | 3 |
| S31 | Yes | Yes | Yes | No | Yes | Yes | Yes | Yes | 7 |
| S32 | Yes | Yes | Yes | Yes | Yes | Yes | Yes | Yes | 8 |
| S33 | Yes | Yes | Yes | No | Yes | Yes | Yes | Yes | 7 |
| S34 | Yes | Yes | Yes | No | Yes | Yes | Yes | Yes | 7 |
| S35 | Yes | Yes | Yes | Yes | Yes | Yes | Yes | Yes | 8 |
| S36 | Yes | Yes | Yes | Yes | Yes | Yes | Yes | Yes | 8 |
| S37 | Yes | No | Yes | Yes | Yes | Yes | Yes | Yes | 7 |
| S38 | Yes | Yes | Yes | No | No | Yes | Yes | Yes | 6 |
| S39 | Yes | Yes | Yes | Yes | Yes | Yes | Yes | Yes | 8 |
| S40 | Yes | Yes | Yes | Yes | Yes | Yes | Yes | Yes | 8 |
| S41 | Yes | Yes | Yes | Yes | No | Yes | Yes | Yes | 7 |
| S42 | Yes | Yes | Yes | Yes | Yes | Yes | Yes | Yes | 8 |
| S43 | No | Yes | Yes | Yes | No | Yes | Yes | Yes | 6 |
| S44 | Yes | Yes | Yes | Yes | Yes | Yes | Yes | Yes | 8 |
| S45 | Yes | Yes | Yes | Yes | No | Yes | Yes | Yes | 7 |
| S46 | Yes | No | Yes | No | No | Yes | Yes | Yes | 5 |
| S47 | Yes | Yes | Yes | No | Yes | Yes | Yes | Yes | 7 |
| S48 | Yes | Yes | Yes | Yes | Yes | Yes | Yes | Yes | 8 |
| S49 | Yes | Yes | Yes | No | Yes | Yes | Yes | Yes | 7 |
| S50 | Yes | Yes | Yes | Yes | Yes | Yes | Yes | Yes | 8 |
| S51 | Yes | Yes | Yes | No | Yes | Yes | Yes | Yes | 7 |
| S52 | Yes | Yes | Yes | No | Yes | Yes | Yes | Yes | 7 |
| S53 | Yes | Yes | Yes | Yes | No | Yes | Yes | Yes | 7 |
| S54 | Yes | Yes | Yes | Yes | Yes | Yes | Yes | Yes | 8 |
| S55 | Yes | Yes | Yes | No | Yes | Yes | Yes | Yes | 7 |
| S56 | Yes | Yes | Yes | Yes | Yes | Yes | Yes | Yes | 8 |
| S57 | Yes | Yes | Yes | Yes | Yes | Yes | Yes | Yes | 8 |
| S58 | Yes | Yes | Yes | Yes | Yes | Yes | Yes | Yes | 8 |
| S59 | Yes | Yes | Yes | Yes | Yes | Yes | Yes | Yes | 8 |
| S60 | Yes | No | Yes | Yes | Yes | Yes | Yes | Yes | 7 |
| S61 | Yes | Yes | Yes | Yes | Yes | Yes | Yes | Yes | 8 |
| S62 | Yes | Yes | Yes | Yes | No | No | No | Yes | 5 |
| S63 | Yes | Yes | Yes | Yes | Yes | Yes | Yes | Yes | 8 |
| S64 | Yes | Yes | Yes | Yes | Yes | Yes | Yes | Yes | 8 |
| S65 | Yes | No | Yes | Yes | No | No | Yes | Yes | 5 |
| S66 | Yes | Yes | Yes | Yes | Yes | Yes | Yes | Yes | 8 |
| S67 | Yes | Yes | Yes | Yes | Yes | Yes | Yes | Yes | 8 |
| S68 | Yes | Yes | Yes | Yes | Yes | Yes | Yes | Yes | 8 |
| S69 | Yes | No | Yes | Yes | No | Yes | Yes | Yes | 6 |
| S70 | Yes | Yes | Yes | Yes | Yes | Yes | Yes | Yes | 8 |
| S71 | Yes | Yes | Yes | Yes | Yes | Yes | Yes | Yes | 8 |
| S72 | Yes | Yes | Yes | Yes | Yes | Yes | Yes | Yes | 8 |
| S73 | Yes | Yes | Yes | Yes | Yes | Yes | Yes | Yes | 8 |
| S74 | Yes | Yes | Yes | Yes | No | Yes | Yes | Yes | 7 |
| S75 | Yes | Yes | Yes | No | No | No | No | Yes | 4 |
| S76 | Yes | Yes | Yes | Yes | Yes | Yes | Yes | Yes | 8 |
| S77 | Yes | Yes | Yes | Yes | Yes | Yes | Yes | Yes | 8 |
| S78 | Yes | Yes | Yes | Yes | Yes | Yes | Yes | Yes | 8 |
| S79 | Yes | Yes | Yes | Yes | No | Yes | Yes | Yes | 7 |
| S80 | Yes | Yes | Yes | Yes | Yes | Yes | Yes | Yes | 8 |
| S81 | Yes | Yes | Yes | Yes | Yes | Yes | Yes | Yes | 8 |
| S82 | Yes | Yes | Yes | Yes | Yes | Yes | Yes | Yes | 8 |
| S83 | Yes | Yes | Yes | Yes | Yes | Yes | Yes | Yes | 8 |
| S84 | Yes | Yes | Yes | Yes | Yes | Yes | Yes | Yes | 8 |
| S85 | Yes | Yes | Yes | Yes | Yes | No | Yes | Yes | 7 |
| S86 | Yes | Yes | Yes | No | Yes | Yes | Yes | Yes | 7 |
| S87 | Yes | Yes | Yes | Yes | Yes | Yes | Yes | Yes | 8 |
| S88 | Yes | Yes | Yes | Yes | Yes | Yes | Yes | Yes | 8 |
| S89 | Yes | Yes | Yes | Yes | Yes | Yes | Yes | Yes | 8 |
| S90 | Yes | Yes | Yes | No | No | Yes | Yes | Yes | 6 |
| S91 | Yes | No | Yes | Yes | No | No | No | Yes | 4 |
| S92 | Yes | Yes | Yes | Yes | No | Yes | Yes | Yes | 7 |
| S93 | Yes | Yes | Yes | Yes | Yes | Yes | Yes | Yes | 8 |
| S94 | Yes | Yes | Yes | Yes | Yes | Yes | Yes | Yes | 8 |
| S95 | Yes | Yes | Yes | Yes | Yes | Yes | Yes | Yes | 8 |
| S96 | Yes | Yes | Yes | Yes | Yes | Yes | Yes | Yes | 8 |
| S97 | Yes | Yes | Yes | Yes | Yes | Yes | Yes | Yes | 8 |
| S98 | Yes | Yes | Yes | Yes | Yes | Yes | Yes | Yes | 8 |
| S99 | Yes | Yes | Yes | Yes | Yes | Yes | Yes | Yes | 8 |
| S100 | Yes | Yes | Yes | Yes | No | Yes | Yes | Yes | 7 |

**Question 1: Were patient’s demographic characteristics clearly described?**

**Question 2: Was the patient’s history clearly described and presented as a timeline?**

**Question 3: Was the current clinical condition of the patient on presentation clearly described?**

**Question 4: Were diagnostic tests or assessment methods and the results clearly described?**

**Question 5: Was the intervention(s) or treatment procedure(s) clearly described?**

**Question 6: Was the post-intervention clinical condition clearly described?**

**Question 7: Were adverse events (harms) or unanticipated events identified and described?**

**Question 8: Does the case report provide takeaway lessons?**

**References**

1. Imm M, Torres LF, Kottapally M. Postoperative delirium in a 64-year-old woman. Cleve Clin J Med. 2017;84(9):690-8.

2. Rodriguez W, Fedorova M, Chand P. Levodopa-Responsive Parkinsonian Syndrome Secondary to a Compressive Craniopharyngioma: A Case Report. Cureus. 2023;15(2):e35621.

3. Isaacson JH. Postoperative confusion in a 42-year-old man. Cleve Clin J Med. 1995;62(6):370-1.

4. Hamlet KM, Pasternak E, Rabai F, Mufti M, Hernaiz Alonso C, Price CC. Perioperative Multidisciplinary Delirium Prevention: A Longitudinal Case Report. A A Pract. 2021;15(1):e01364.

5. Hammoud A, Saade E, Jarry S, Baelen S, Couture EJ, Beaubien-Souligny W, et al. Pulsatile Femoral Vein Doppler and Congestive Delirium, What Is the Relationship?: A Case Report. A A Pract. 2022;16(10):e01627.

6. Hiranuma W, Murata Y, Matsuoka T, Minagawa T, Shimizu T, Kawamoto S. Non-occlusive mesenteric ischemia after trans-catheter aortic valve implantation with thyroid storm: A case report. J Cardiol Cases. 2023;27(1):19-22.

7. Kim JH, Lee SC, Nam CH, Kim T, Ahn HS, Baek JH. Anterior dislocation of a total knee arthroplasty in a patient with postoperative delirium: a case report. Clin Case Rep. 2021;9(11):e05087.

8. Burfeind KG, Tirado Navales AA, Togioka BM, Schenning K. Prevention of postoperative delirium through the avoidance of potentially inappropriate medications in a geriatric surgical patient. BMJ Case Rep. 2021;14(4).

9. Kinoshita H, Kushikata T, Takekawa D, Hirota K. Perioperative abnormal electroencephalography in a later-stage elderly with septic shock: a case report. JA Clin Rep. 2021;7(1):5.

10. Wang Z, Yang Y, Chen Y, Lu K, Chen B. Emergence Delirium in a 29-Year-Old Man following an Uneventful Appendectomy. Case Rep Med. 2021;2021:1338823.

11. Partownavid P, Wang L, Alaei S, Rahman S. Post-dural puncture headache following lumbar spinal drain: an atypical presentation with cognitive symptoms. Anaesth Rep. 2021;9(2):e12127.

12. Ledford CK, VanWagner MJ, Sherman CE, Torp KD. Immersive Virtual Reality Used as Adjunct Anesthesia for Conversion Total Hip Arthroplasty in a 100-Year-Old Patient. Arthroplast Today. 2021;10:149-53.

13. Belanger K, Grassia F, Kortz MW, Thompson JA, DeStefano S, Ojemann S. Management of post-operative delirium following stereoelectroencephalography electrode placement for drug resistant epilepsy: Lessons learned from two case reports. Epilepsy Behav Rep. 2021;16:100438.

14. Bonomo G, Caldiroli D, Bonomo R, Pugliese R, DiMeco F, Zoia C. Reactivation of COVID-19 in a neurosurgical patient with early neuropsychiatric presentation. Does seroconversion mean immunity? Surg Neurol Int. 2021;12:166.

15. Calderone A, Chauvette V, Demers P, Lamarche Y. Frozen Elephant Trunk Repair for Acute Type A Dissection in Right Aortic Arch. Ann Thorac Surg. 2022;113(6):e429-e31.

16. Tashiro R, Fujimura M, Nishizawa T, Saito A, Tominaga T. Cerebral Hyperperfusion and Concomitant Reversible Lesion at the Splenium after Direct Revascularization Surgery for Adult Moyamoya Disease: Possible Involvement of MERS and Watershed Shift Phenomenon. NMC Case Rep J. 2021;8(1):451-6.

17. Gaetano R, De Filippis R, Caroleo M, Segura-Garcia C, De Fazio P. Postoperative heroin-withdrawal delirium treated with clonazepam after urgent cardiac surgery: a case report. Riv Psichiatr. 2020;55(6):366-70.

18. Lim DJ. Intoxication by hand sanitizer due to delirium after infectious spondylitis surgery during the COVID-19 pandemic: A case report and literature review. Int J Surg Case Rep. 2020;77:76-9.

19. Gutman DA, Hassid M, Jeanes Z, Redding AT, Romeo D. Prophylactic Physostigmine for Extreme and Refractory Adult Emergence Delirium, Aimed at Increasing Patient Safety and Reducing Health Care Workplace Violence: A Case Report. A A Pract. 2020;14(6):e01205.

20. El-Shakankery KH, Mieiro L. A key role for comprehensive geriatric assessment in aortic valve replacement. BMJ Case Rep. 2020;13(12).

21. Liu Z, Li R, Wang S, Zhou Y, Yin L, Qu Y, et al. Postoperative delirium after hysteroscopy in young woman: A case report. Medicine (Baltimore). 2019;98(44):e17663.

22. Levantesi L, De Cosmo G, Logroscino G, Saracco M. Recurrent postoperative delirium in spinocerebellar ataxia type 2: a case report. J Med Case Rep. 2019;13(1):112.

23. Muller MD, Capp AM, Hill J, Hoffer A, Otworth JR, McQuillan PM, et al. Anesthetic Management of Elderly Patients With Down Syndrome: A Case Report. J Perianesth Nurs. 2020;35(3):243-9.

24. Chernyak Y, Teh L. Medically Induced Exacerbation of PTSD Following Lung Transplantation: A Case Series. J Clin Psychol Med Settings. 2020;27(2):305-9.

25. Zhu J, Al-Alkim F, Hussaini T, Vertinsky A, Byrne D, Erb SR, et al. Occult central pontine myelinolysis post liver transplant: A consequence of pre-transplant hyponatremia. Ann Hepatol. 2019;18(4):651-4.

26. Akelma H, Kilic ET, Salik F, Bicak EA, Kaya S. Postoperative cognitive dysfunction developed in donor nephrectomy- Case report. Niger J Clin Pract. 2019;22(6):877-80.

27. Yu H, Shen X. Postoperative delirium after partial laryngectomy in a middle-aged patient: A case report. Medicine (Baltimore). 2018;97(8):e9988.

28. Kamienski MC, McCartney MA, McLaughlin M, Pallaria T. Pediatric Emergence Delirium: A Case Study. J Perianesth Nurs. 2019;34(3):469-75.

29. Khazi FM, Al-Safadi F, Al Asaad MMR, Aljassim O. Is baseline cerebral oximetry a better predictor than carotid scan for postoperative delirium in cardiac surgery? J Saudi Heart Assoc. 2018;30(3):260-3.

30. Winterberg V, Cnyrim CD, Reuter S. Severe Delirium after Combined Pancreas and Kidney Transplantation. Dtsch Arztebl Int. 2018;115(27-28):476.

31. Acevedo FA, Kim EJ, Chyatte DA, Nielsen VG. Rare cause of delirium and hypoxemia after coronary bypass surgery: transdermal lidocaine patch-associated methemoglobinemia. Int J Legal Med. 2018;132(3):767-9.

32. Read MD, Maani CV, Blackwell S. Dexmedetomidine as a Rescue Therapy for Emergence Delirium in Adults: A Case Series. A A Case Rep. 2017;9(1):20-3.

33. Mitchell A, Marquis F. Can takotsubo cardiomyopathy be diagnosed by autopsy? Report of a presumed case presenting as cardiac rupture. BMC Clin Pathol. 2017;17:4.

34. Santos CD, Ratzlaff RA, Meder JC, Atwal PS, Joyce NE. Ornithine Transcarbamylase Deficiency: If at First You Do Not Diagnose, Try and Try Again. Case Rep Crit Care. 2017;2017:8724810.

35. Benhamou D, Brouquet A. Postoperative cerebral dysfunction in the elderly: Diagnosis and prophylaxis. J Visc Surg. 2016;153(6s):S27-s32.

36. da Costa FH, Herrera PA, Pereira-Stabile CL, Vitti Stabile GA. Postoperative Delirium Following Orthognathic Surgery in a Young Patient. J Oral Maxillofac Surg. 2017;75(2):284.e1-.e4.

37. Nag DS, Chatterjee A, Samaddar DP, Singh H. Sepsis associated delirium mimicking postoperative delirium as the initial presenting symptom of urosepsis in a patient who underwent nephrolithotomy. World J Clin Cases. 2016;4(5):130-4.

38. Brown GD, Muzyk AJ, Preud'homme XA. Prolonged Delirium With Catatonia Following Orthotopic Liver Transplant Responsive to Memantine. J Psychiatr Pract. 2016;22(2):128-32.

39. Drobish JK, Kelz MB, DiPuppo PM, Cook-Sather SD. Emergence delirium with transient associative agnosia and expressive aphasia reversed by flumazenil in a pediatric patient. A A Case Rep. 2015;4(11):148-50.

40. Moll V, Ward CT, Zivot JB. Antipsychotic-Induced Neuroleptic Malignant Syndrome After Cardiac Surgery. A A Case Rep. 2016;7(1):5-8.

41. Waked WJ, Gordon RM, Whiteson JH, Baron EM. Recognizing encephalopathy and delirium in the cardiopulmonary rehabilitation setting. Rehabil Psychol. 2015;60(2):201-10.

42. Whalin MK, Kreuzer M, Halenda KM, García PS. Missed Opportunities for Intervention in a Patient With Prolonged Postoperative Delirium. Clin Ther. 2015;37(12):2706-10.

43. Liu JJ, Dahlin BC, Waldau B. Contrast encephalopathy after coiling in the setting of obstructive sleep apnoea. BMJ Case Rep. 2015;2015.

44. Aksakal N, Erçetin C, Özçınar B, Aral F, Erbil Y. Lithium-associated primary hyperparathyroidism complicated by nephrogenic diabetes insipidus. Ulus Cerrahi Derg. 2015;31(3):166-9.

45. Sorrell JM. Postoperative cognitive dysfunction in older adults: a call for nursing involvement. J Psychosoc Nurs Ment Health Serv. 2014;52(11):17-20.

46. Bican Demir A, Erer Özbek S, Bora I, Hakyemez B, Tırnova I, Kaya E. Two Cases With Developing Neurologic Complications After Liver Transplant. Exp Clin Transplant. 2016;14(6):685-7.

47. Tattersall TL, Thangasamy IA, Reynolds J. Bilateral adrenal haemorrhage associated with heparin-induced thrombocytopaenia during treatment of Fournier gangrene. BMJ Case Rep. 2014;2014.

48. Marcantonio ER. Postoperative delirium: a 76-year-old woman with delirium following surgery. Jama. 2012;308(1):73-81.

49. Licht E, Siegler EL, Reid MC. Can the cognitively impaired safely use patient-controlled analgesia? J Opioid Manag. 2009;5(5):307-12.

50. Lithium: Nephrogenic diabetes insipidus, hypernatraemia and delirium: case report. Reactions Weekly. 2013(1468):28.

51. Gumber D, Rodin M, Wildes TM. Postradiation osteosarcoma in an older prostate cancer survivor: case study and literature review with emphasis on geriatric principles. Case Rep Oncol. 2013;6(2):250-5.

52. Fagenholz PJ, Bowler GM, Carnochan FM, Walker WS. Systemic local anaesthetic toxicity from continuous thoracic paravertebral block. Br J Anaesth. 2012;109(2):260-2.

53. Habunaga H, Nakamura H. Intracranial subdural hematoma as a cause of postoperative delirium and headache in cervical laminoplasty: A case report and review of the literature. Sas j. 2011;5(1):1-3.

54. Madden K, Turkel S, Jacobson J, Epstein D, Moromisato DY. Recurrent delirium after surgery for congenital heart disease in an infant. Pediatr Crit Care Med. 2011;12(6):e413-5.

55. Crane JH, Suda KJ. Oxycodone induced delirium and agitation in an elderly patient following total right knee arthroplasty. Int J Clin Pharm. 2011;33(5):733-6.

56. Cavallini M, Saracco MG, Aguggia M. Post operative delirium with hyponatriemia after transurethral resection of the prostate: a case of transurethral resection syndrome? Acta Neurol Belg. 2011;111(2):152-4.

57. Nomoto K, Scurlock C, Bronster D. Dexmedetomidine controls twitch-convulsive syndrome in the course of uremic encephalopathy. J Clin Anesth. 2011;23(8):646-8.

58. Mantz J, Hemmings HC, Jr., Boddaert J. Case scenario: postoperative delirium in elderly surgical patients. Anesthesiology. 2010;112(1):189-95.

59. Saito S, Kobayashi T, Osawa T, Kato S. Effectiveness of Japanese herbal medicine yokukansan for alleviating psychiatric symptoms after traumatic brain injury. Psychogeriatrics. 2010;10(1):45-8.

60. Özdemir B, Çelik C, Çinar A, Özşahin A. Relief by electroconvulsive therapy for postsurgical delirium in malignant catatonia. J ect. 2010;26(4):272-3.

61. Sieber FE. Postoperative delirium in the elderly surgical patient. Anesthesiol Clin. 2009;27(3):451-64, table of contents.

62. Mauck KF, Litin SC. Clinical Pearls in perioperative medicine. Mayo Clin Proc. 2009;84(6):546-50.

63. Reich M, Rohn R, Lefevre D. Surgical intensive care unit (ICU) delirium: a "psychosomatic" problem? Palliat Support Care. 2010;8(2):221-5.

64. Arai YC, Ito A, Hibino S, Niwa S, Ueda W. Auricular Acupunctures are Effective for the Prevention of Postoperative Agitation in Old Patients. Evid Based Complement Alternat Med. 2010;7(3):383-6.

65. Saniova B, Drobny M, Sulaj M. Delirium and postoperative cognitive dysfunction after general anesthesia. Medical Science Monitor. 2009;15(5):CS81-7.

66. Fricchione GL, Nejad SH, Esses JA, Cummings TJ, Jr., Querques J, Cassem NH, et al. Postoperative delirium. Am J Psychiatry. 2008;165(7):803-12.

67. Joaquim AF, Shaffrey CC, Sansur CA, Shaffrey CI. Man-in-the-barrel syndrome after thoracoilium fusion. J Neurosurg Spine. 2008;9(6):566-9.

68. Heidrich DE. Delirium: an under-recognized problem. Clin J Oncol Nurs. 2007;11(6):805-7.

69. Gillis AJ, MacDonald B. Unmasking delirium. Can Nurse. 2006;102(9):18-24.

70. Mohri-Ikuzawa YDDSP, Inada HD, Takahashi NDDSP, Kohase HDDSP, Jinno SDDSP, Umino MDDSP. Delirium During Intravenous Sedation With Midazolam Alone and With Propofol in Dental Treatment. Anesthesia Progress. 2006;53(3):95-7.

71. Onishi H, Sugimasa Y, Kawanishi C, Onose M. Wernicke encephalopathy presented in the form of postoperative delirium in a patient with hepatocellular carcinoma and liver cirrhosis: a case report and review of the literature. Palliat Support Care. 2005;3(4):337-40.

72. Ogasawara K, Komoribayashi N, Kobayashi M, Fukuda T, Inoue T, Yamadate K, et al. Neural damage caused by cerebral hyperperfusion after arterial bypass surgery in a patient with moyamoya disease: case report. Neurosurgery. 2005;56(6):E1380; discussion E.

73. Okumura K. Risperidone therapy for post-operative delirium in elderly patients. Psychogeriatrics. 2005;5(3):108-11.

74. Kobayashi K, Higashima M, Mutou K, Kidani T, Tachibana O, Yamashita J, et al. Severe delirium due to basal forebrain vascular lesion and efficacy of donepezil. Prog Neuropsychopharmacol Biol Psychiatry. 2004;28(7):1189-94.

75. Audu PB, Curtis N, Armstead V. An unusual case of emergence delirium. J Clin Anesth. 2004;16(7):545-7.

76. Burns SM. Delirium during emergence from anesthesia: a case study. Crit Care Nurse. 2003;23(1):66-9.

77. Wendel I. A case study of postoperative delirium. Aorn j. 2002;75(3):595-600.

78. Hanania M, Kitain E. Melatonin for treatment and prevention of postoperative delirium. Anesth Analg. 2002;94(2):338-9, table of contents.

79. Harris RA, Poole A. Beware of bismuth: post maxillectomy delirium. ANZ J Surg. 2002;72(11):846-7.

80. Torres R, Mittal D, Kennedy R. Use of quetiapine in delirium: case reports. Psychosomatics. 2001;42(4):347-9.

81. Gerrah R, Abramovitch Y, Elami A. Traumatic memory: a cause for postoperative delirium--a diagnostic dilemma. Isr Med Assoc J. 2001;3(11):858-9.

82. McLoughlin PD. Pethidine reverses morphine-induced delirium. Anaesth Intensive Care. 2000;28(3):311-2.

83. Perrault LP, Denault AY, Carrier M, Cartier R, Bélisle S. Torsades de pointes secondary to intravenous haloperidol after coronary bypass grafting surgery. Can J Anaesth. 2000;47(3):251-4.

84. Stanford BJ, Stanford SC. Postoperative delirium indicating an adverse drug interaction involving the selective serotonin reuptake inhibitor, paroxetine? J Psychopharmacol. 1999;13(3):313-7.

85. Wells LT, Rasch DK. Emergence "delirium" after sevoflurane anesthesia: a paranoid delusion? Anesth Analg. 1999;88(6):1308-10.

86. Nakata Y, Kimura K, Tomioka N, Kawasaki S, Takagaki Y. Successful simultaneous operation of concomitant early gastric cancer, transverse colon cancer, and a common iliac artery aneurysm. Surg Today. 1999;29(8):782-4.

87. Yamasaki K, Morimoto N, Gion T, Yanaga K. Delirium and a subclavian abscess. Lancet. 1997;350(9087):1294.

88. Eden BM, Foreman MD. Problems associated with underrecognition of delirium in critical care: a case study. Heart Lung. 1996;25(5):388-400.

89. Levenson JL. High-dose intravenous haloperidol for agitated delirium following lung transplantation. Psychosomatics. 1995;36(1):66-8.

90. Sekimoto M, Hayasaka S, Noda S, Iijima M, Setogawa T. Psychiatric complications after ocular surgery. Ophthalmologica. 1993;206(3):113-4.

91. Gullahorn GM, Bohman HR, Wallace MR. Anaesthesia emergence delirium after mefloquine prophylaxis. Lancet. 1993;341(8845):632.

92. Asada T, Kobayashi M, Hirano M, Atobe M. An autopsy case of coexisting portal systemic encephalopathy and senile dementia of the Alzheimer type. Jpn J Psychiatry Neurol. 1993;47(3):651-6.

93. Olympio MA. Postanesthetic delirium: historical perspectives. J Clin Anesth. 1991;3(1):60-3.

94. Freiberger JJ, Marsicano TH. Alprazolam withdrawal presenting as delirium after cardiac surgery. J Cardiothorac Vasc Anesth. 1991;5(2):150-2.

95. Craven JL. Cyclosporine-associated organic mental disorders in liver transplant recipients. Psychosomatics. 1991;32(1):94-102.

96. Weinger MB, Swerdlow NR, Millar WL. Acute postoperative delirium and extrapyramidal signs in a previously healthy parturient. Anesth Analg. 1988;67(3):291-5.

97. Magni G, De Leo D. Complex management of postcardiotomy delirium. South Med J. 1983;76(1):94-5.

98. Wolpert EA, Margul B, Replogle R, Sheinin JC. Coronary artery bypass in a patient on lithium carbonate prophylaxis. J Nerv Ment Dis. 1982;170(3):181-4.

99. Blitt CD, Petty WC. Reversal of lorazepam delirium by physostigmine. Anesth Analg. 1975;54(5):607-8.

100. Galdston R. Psychotic reaction to the success of cardiac valvotomy: a case report. Psychiatry Med. 1970;1(4):367-73.

**References**

1. Imm M, Torres LF, Kottapally M. Postoperative delirium in a 64-year-old woman. Cleve Clin J Med. 2017;84(9):690-8.

2. Rodriguez W, Fedorova M, Chand P. Levodopa-Responsive Parkinsonian Syndrome Secondary to a Compressive Craniopharyngioma: A Case Report. Cureus. 2023;15(2):e35621.

3. Isaacson JH. Postoperative confusion in a 42-year-old man. Cleve Clin J Med. 1995;62(6):370-1.

4. Hamlet KM, Pasternak E, Rabai F, Mufti M, Hernaiz Alonso C, Price CC. Perioperative Multidisciplinary Delirium Prevention: A Longitudinal Case Report. A A Pract. 2021;15(1):e01364.

5. Hammoud A, Saade E, Jarry S, Baelen S, Couture EJ, Beaubien-Souligny W, et al. Pulsatile Femoral Vein Doppler and Congestive Delirium, What Is the Relationship?: A Case Report. A A Pract. 2022;16(10):e01627.

6. Hiranuma W, Murata Y, Matsuoka T, Minagawa T, Shimizu T, Kawamoto S. Non-occlusive mesenteric ischemia after trans-catheter aortic valve implantation with thyroid storm: A case report. J Cardiol Cases. 2023;27(1):19-22.

7. Kim JH, Lee SC, Nam CH, Kim T, Ahn HS, Baek JH. Anterior dislocation of a total knee arthroplasty in a patient with postoperative delirium: a case report. Clin Case Rep. 2021;9(11):e05087.

8. Burfeind KG, Tirado Navales AA, Togioka BM, Schenning K. Prevention of postoperative delirium through the avoidance of potentially inappropriate medications in a geriatric surgical patient. BMJ Case Rep. 2021;14(4).

9. Kinoshita H, Kushikata T, Takekawa D, Hirota K. Perioperative abnormal electroencephalography in a later-stage elderly with septic shock: a case report. JA Clin Rep. 2021;7(1):5.

10. Wang Z, Yang Y, Chen Y, Lu K, Chen B. Emergence Delirium in a 29-Year-Old Man following an Uneventful Appendectomy. Case Rep Med. 2021;2021:1338823.

11. Partownavid P, Wang L, Alaei S, Rahman S. Post-dural puncture headache following lumbar spinal drain: an atypical presentation with cognitive symptoms. Anaesth Rep. 2021;9(2):e12127.

12. Ledford CK, VanWagner MJ, Sherman CE, Torp KD. Immersive Virtual Reality Used as Adjunct Anesthesia for Conversion Total Hip Arthroplasty in a 100-Year-Old Patient. Arthroplast Today. 2021;10:149-53.

13. Belanger K, Grassia F, Kortz MW, Thompson JA, DeStefano S, Ojemann S. Management of post-operative delirium following stereoelectroencephalography electrode placement for drug resistant epilepsy: Lessons learned from two case reports. Epilepsy Behav Rep. 2021;16:100438.

14. Bonomo G, Caldiroli D, Bonomo R, Pugliese R, DiMeco F, Zoia C. Reactivation of COVID-19 in a neurosurgical patient with early neuropsychiatric presentation. Does seroconversion mean immunity? Surg Neurol Int. 2021;12:166.

15. Calderone A, Chauvette V, Demers P, Lamarche Y. Frozen Elephant Trunk Repair for Acute Type A Dissection in Right Aortic Arch. Ann Thorac Surg. 2022;113(6):e429-e31.

16. Tashiro R, Fujimura M, Nishizawa T, Saito A, Tominaga T. Cerebral Hyperperfusion and Concomitant Reversible Lesion at the Splenium after Direct Revascularization Surgery for Adult Moyamoya Disease: Possible Involvement of MERS and Watershed Shift Phenomenon. NMC Case Rep J. 2021;8(1):451-6.

17. Gaetano R, De Filippis R, Caroleo M, Segura-Garcia C, De Fazio P. Postoperative heroin-withdrawal delirium treated with clonazepam after urgent cardiac surgery: a case report. Riv Psichiatr. 2020;55(6):366-70.

18. Lim DJ. Intoxication by hand sanitizer due to delirium after infectious spondylitis surgery during the COVID-19 pandemic: A case report and literature review. Int J Surg Case Rep. 2020;77:76-9.

19. Gutman DA, Hassid M, Jeanes Z, Redding AT, Romeo D. Prophylactic Physostigmine for Extreme and Refractory Adult Emergence Delirium, Aimed at Increasing Patient Safety and Reducing Health Care Workplace Violence: A Case Report. A A Pract. 2020;14(6):e01205.

20. El-Shakankery KH, Mieiro L. A key role for comprehensive geriatric assessment in aortic valve replacement. BMJ Case Rep. 2020;13(12).

21. Liu Z, Li R, Wang S, Zhou Y, Yin L, Qu Y, et al. Postoperative delirium after hysteroscopy in young woman: A case report. Medicine (Baltimore). 2019;98(44):e17663.

22. Levantesi L, De Cosmo G, Logroscino G, Saracco M. Recurrent postoperative delirium in spinocerebellar ataxia type 2: a case report. J Med Case Rep. 2019;13(1):112.

23. Muller MD, Capp AM, Hill J, Hoffer A, Otworth JR, McQuillan PM, et al. Anesthetic Management of Elderly Patients With Down Syndrome: A Case Report. J Perianesth Nurs. 2020;35(3):243-9.

24. Chernyak Y, Teh L. Medically Induced Exacerbation of PTSD Following Lung Transplantation: A Case Series. J Clin Psychol Med Settings. 2020;27(2):305-9.

25. Zhu J, Al-Alkim F, Hussaini T, Vertinsky A, Byrne D, Erb SR, et al. Occult central pontine myelinolysis post liver transplant: A consequence of pre-transplant hyponatremia. Ann Hepatol. 2019;18(4):651-4.

26. Akelma H, Kilic ET, Salik F, Bicak EA, Kaya S. Postoperative cognitive dysfunction developed in donor nephrectomy- Case report. Niger J Clin Pract. 2019;22(6):877-80.

27. Yu H, Shen X. Postoperative delirium after partial laryngectomy in a middle-aged patient: A case report. Medicine (Baltimore). 2018;97(8):e9988.

28. Kamienski MC, McCartney MA, McLaughlin M, Pallaria T. Pediatric Emergence Delirium: A Case Study. J Perianesth Nurs. 2019;34(3):469-75.

29. Khazi FM, Al-Safadi F, Al Asaad MMR, Aljassim O. Is baseline cerebral oximetry a better predictor than carotid scan for postoperative delirium in cardiac surgery? J Saudi Heart Assoc. 2018;30(3):260-3.

30. Winterberg V, Cnyrim CD, Reuter S. Severe Delirium after Combined Pancreas and Kidney Transplantation. Dtsch Arztebl Int. 2018;115(27-28):476.

31. Acevedo FA, Kim EJ, Chyatte DA, Nielsen VG. Rare cause of delirium and hypoxemia after coronary bypass surgery: transdermal lidocaine patch-associated methemoglobinemia. Int J Legal Med. 2018;132(3):767-9.

32. Read MD, Maani CV, Blackwell S. Dexmedetomidine as a Rescue Therapy for Emergence Delirium in Adults: A Case Series. A A Case Rep. 2017;9(1):20-3.

33. Mitchell A, Marquis F. Can takotsubo cardiomyopathy be diagnosed by autopsy? Report of a presumed case presenting as cardiac rupture. BMC Clin Pathol. 2017;17:4.

34. Santos CD, Ratzlaff RA, Meder JC, Atwal PS, Joyce NE. Ornithine Transcarbamylase Deficiency: If at First You Do Not Diagnose, Try and Try Again. Case Rep Crit Care. 2017;2017:8724810.

35. Benhamou D, Brouquet A. Postoperative cerebral dysfunction in the elderly: Diagnosis and prophylaxis. J Visc Surg. 2016;153(6s):S27-s32.

36. da Costa FH, Herrera PA, Pereira-Stabile CL, Vitti Stabile GA. Postoperative Delirium Following Orthognathic Surgery in a Young Patient. J Oral Maxillofac Surg. 2017;75(2):284.e1-.e4.

37. Nag DS, Chatterjee A, Samaddar DP, Singh H. Sepsis associated delirium mimicking postoperative delirium as the initial presenting symptom of urosepsis in a patient who underwent nephrolithotomy. World J Clin Cases. 2016;4(5):130-4.

38. Brown GD, Muzyk AJ, Preud'homme XA. Prolonged Delirium With Catatonia Following Orthotopic Liver Transplant Responsive to Memantine. J Psychiatr Pract. 2016;22(2):128-32.

39. Drobish JK, Kelz MB, DiPuppo PM, Cook-Sather SD. Emergence delirium with transient associative agnosia and expressive aphasia reversed by flumazenil in a pediatric patient. A A Case Rep. 2015;4(11):148-50.

40. Moll V, Ward CT, Zivot JB. Antipsychotic-Induced Neuroleptic Malignant Syndrome After Cardiac Surgery. A A Case Rep. 2016;7(1):5-8.

41. Waked WJ, Gordon RM, Whiteson JH, Baron EM. Recognizing encephalopathy and delirium in the cardiopulmonary rehabilitation setting. Rehabil Psychol. 2015;60(2):201-10.

42. Whalin MK, Kreuzer M, Halenda KM, García PS. Missed Opportunities for Intervention in a Patient With Prolonged Postoperative Delirium. Clin Ther. 2015;37(12):2706-10.

43. Liu JJ, Dahlin BC, Waldau B. Contrast encephalopathy after coiling in the setting of obstructive sleep apnoea. BMJ Case Rep. 2015;2015.

44. Aksakal N, Erçetin C, Özçınar B, Aral F, Erbil Y. Lithium-associated primary hyperparathyroidism complicated by nephrogenic diabetes insipidus. Ulus Cerrahi Derg. 2015;31(3):166-9.

45. Sorrell JM. Postoperative cognitive dysfunction in older adults: a call for nursing involvement. J Psychosoc Nurs Ment Health Serv. 2014;52(11):17-20.

46. Bican Demir A, Erer Özbek S, Bora I, Hakyemez B, Tırnova I, Kaya E. Two Cases With Developing Neurologic Complications After Liver Transplant. Exp Clin Transplant. 2016;14(6):685-7.

47. Tattersall TL, Thangasamy IA, Reynolds J. Bilateral adrenal haemorrhage associated with heparin-induced thrombocytopaenia during treatment of Fournier gangrene. BMJ Case Rep. 2014;2014.

48. Marcantonio ER. Postoperative delirium: a 76-year-old woman with delirium following surgery. Jama. 2012;308(1):73-81.

49. Licht E, Siegler EL, Reid MC. Can the cognitively impaired safely use patient-controlled analgesia? J Opioid Manag. 2009;5(5):307-12.

50. Lithium: Nephrogenic diabetes insipidus, hypernatraemia and delirium: case report. Reactions Weekly. 2013(1468):28.

51. Gumber D, Rodin M, Wildes TM. Postradiation osteosarcoma in an older prostate cancer survivor: case study and literature review with emphasis on geriatric principles. Case Rep Oncol. 2013;6(2):250-5.

52. Fagenholz PJ, Bowler GM, Carnochan FM, Walker WS. Systemic local anaesthetic toxicity from continuous thoracic paravertebral block. Br J Anaesth. 2012;109(2):260-2.

53. Habunaga H, Nakamura H. Intracranial subdural hematoma as a cause of postoperative delirium and headache in cervical laminoplasty: A case report and review of the literature. Sas j. 2011;5(1):1-3.

54. Madden K, Turkel S, Jacobson J, Epstein D, Moromisato DY. Recurrent delirium after surgery for congenital heart disease in an infant. Pediatr Crit Care Med. 2011;12(6):e413-5.

55. Crane JH, Suda KJ. Oxycodone induced delirium and agitation in an elderly patient following total right knee arthroplasty. Int J Clin Pharm. 2011;33(5):733-6.

56. Cavallini M, Saracco MG, Aguggia M. Post operative delirium with hyponatriemia after transurethral resection of the prostate: a case of transurethral resection syndrome? Acta Neurol Belg. 2011;111(2):152-4.

57. Nomoto K, Scurlock C, Bronster D. Dexmedetomidine controls twitch-convulsive syndrome in the course of uremic encephalopathy. J Clin Anesth. 2011;23(8):646-8.

58. Mantz J, Hemmings HC, Jr., Boddaert J. Case scenario: postoperative delirium in elderly surgical patients. Anesthesiology. 2010;112(1):189-95.

59. Saito S, Kobayashi T, Osawa T, Kato S. Effectiveness of Japanese herbal medicine yokukansan for alleviating psychiatric symptoms after traumatic brain injury. Psychogeriatrics. 2010;10(1):45-8.

60. Özdemir B, Çelik C, Çinar A, Özşahin A. Relief by electroconvulsive therapy for postsurgical delirium in malignant catatonia. J ect. 2010;26(4):272-3.

61. Sieber FE. Postoperative delirium in the elderly surgical patient. Anesthesiol Clin. 2009;27(3):451-64, table of contents.

62. Mauck KF, Litin SC. Clinical Pearls in perioperative medicine. Mayo Clin Proc. 2009;84(6):546-50.

63. Reich M, Rohn R, Lefevre D. Surgical intensive care unit (ICU) delirium: a "psychosomatic" problem? Palliat Support Care. 2010;8(2):221-5.

64. Arai YC, Ito A, Hibino S, Niwa S, Ueda W. Auricular Acupunctures are Effective for the Prevention of Postoperative Agitation in Old Patients. Evid Based Complement Alternat Med. 2010;7(3):383-6.

65. Saniova B, Drobny M, Sulaj M. Delirium and postoperative cognitive dysfunction after general anesthesia. Medical Science Monitor. 2009;15(5):CS81-7.

66. Fricchione GL, Nejad SH, Esses JA, Cummings TJ, Jr., Querques J, Cassem NH, et al. Postoperative delirium. Am J Psychiatry. 2008;165(7):803-12.

67. Joaquim AF, Shaffrey CC, Sansur CA, Shaffrey CI. Man-in-the-barrel syndrome after thoracoilium fusion. J Neurosurg Spine. 2008;9(6):566-9.

68. Heidrich DE. Delirium: an under-recognized problem. Clin J Oncol Nurs. 2007;11(6):805-7.

69. Gillis AJ, MacDonald B. Unmasking delirium. Can Nurse. 2006;102(9):18-24.

70. Mohri-Ikuzawa YDDSP, Inada HD, Takahashi NDDSP, Kohase HDDSP, Jinno SDDSP, Umino MDDSP. Delirium During Intravenous Sedation With Midazolam Alone and With Propofol in Dental Treatment. Anesthesia Progress. 2006;53(3):95-7.

71. Onishi H, Sugimasa Y, Kawanishi C, Onose M. Wernicke encephalopathy presented in the form of postoperative delirium in a patient with hepatocellular carcinoma and liver cirrhosis: a case report and review of the literature. Palliat Support Care. 2005;3(4):337-40.

72. Ogasawara K, Komoribayashi N, Kobayashi M, Fukuda T, Inoue T, Yamadate K, et al. Neural damage caused by cerebral hyperperfusion after arterial bypass surgery in a patient with moyamoya disease: case report. Neurosurgery. 2005;56(6):E1380; discussion E.

73. Okumura K. Risperidone therapy for post-operative delirium in elderly patients. Psychogeriatrics. 2005;5(3):108-11.

74. Kobayashi K, Higashima M, Mutou K, Kidani T, Tachibana O, Yamashita J, et al. Severe delirium due to basal forebrain vascular lesion and efficacy of donepezil. Prog Neuropsychopharmacol Biol Psychiatry. 2004;28(7):1189-94.

75. Audu PB, Curtis N, Armstead V. An unusual case of emergence delirium. J Clin Anesth. 2004;16(7):545-7.

76. Burns SM. Delirium during emergence from anesthesia: a case study. Crit Care Nurse. 2003;23(1):66-9.

77. Wendel I. A case study of postoperative delirium. Aorn j. 2002;75(3):595-600.

78. Hanania M, Kitain E. Melatonin for treatment and prevention of postoperative delirium. Anesth Analg. 2002;94(2):338-9, table of contents.

79. Harris RA, Poole A. Beware of bismuth: post maxillectomy delirium. ANZ J Surg. 2002;72(11):846-7.

80. Torres R, Mittal D, Kennedy R. Use of quetiapine in delirium: case reports. Psychosomatics. 2001;42(4):347-9.

81. Gerrah R, Abramovitch Y, Elami A. Traumatic memory: a cause for postoperative delirium--a diagnostic dilemma. Isr Med Assoc J. 2001;3(11):858-9.

82. McLoughlin PD. Pethidine reverses morphine-induced delirium. Anaesth Intensive Care. 2000;28(3):311-2.

83. Perrault LP, Denault AY, Carrier M, Cartier R, Bélisle S. Torsades de pointes secondary to intravenous haloperidol after coronary bypass grafting surgery. Can J Anaesth. 2000;47(3):251-4.

84. Stanford BJ, Stanford SC. Postoperative delirium indicating an adverse drug interaction involving the selective serotonin reuptake inhibitor, paroxetine? J Psychopharmacol. 1999;13(3):313-7.

85. Wells LT, Rasch DK. Emergence "delirium" after sevoflurane anesthesia: a paranoid delusion? Anesth Analg. 1999;88(6):1308-10.

86. Nakata Y, Kimura K, Tomioka N, Kawasaki S, Takagaki Y. Successful simultaneous operation of concomitant early gastric cancer, transverse colon cancer, and a common iliac artery aneurysm. Surg Today. 1999;29(8):782-4.

87. Yamasaki K, Morimoto N, Gion T, Yanaga K. Delirium and a subclavian abscess. Lancet. 1997;350(9087):1294.

88. Eden BM, Foreman MD. Problems associated with underrecognition of delirium in critical care: a case study. Heart Lung. 1996;25(5):388-400.

89. Levenson JL. High-dose intravenous haloperidol for agitated delirium following lung transplantation. Psychosomatics. 1995;36(1):66-8.

90. Sekimoto M, Hayasaka S, Noda S, Iijima M, Setogawa T. Psychiatric complications after ocular surgery. Ophthalmologica. 1993;206(3):113-4.

91. Gullahorn GM, Bohman HR, Wallace MR. Anaesthesia emergence delirium after mefloquine prophylaxis. Lancet. 1993;341(8845):632.

92. Asada T, Kobayashi M, Hirano M, Atobe M. An autopsy case of coexisting portal systemic encephalopathy and senile dementia of the Alzheimer type. Jpn J Psychiatry Neurol. 1993;47(3):651-6.

93. Olympio MA. Postanesthetic delirium: historical perspectives. J Clin Anesth. 1991;3(1):60-3.

94. Freiberger JJ, Marsicano TH. Alprazolam withdrawal presenting as delirium after cardiac surgery. J Cardiothorac Vasc Anesth. 1991;5(2):150-2.

95. Craven JL. Cyclosporine-associated organic mental disorders in liver transplant recipients. Psychosomatics. 1991;32(1):94-102.

96. Weinger MB, Swerdlow NR, Millar WL. Acute postoperative delirium and extrapyramidal signs in a previously healthy parturient. Anesth Analg. 1988;67(3):291-5.

97. Magni G, De Leo D. Complex management of postcardiotomy delirium. South Med J. 1983;76(1):94-5.

98. Wolpert EA, Margul B, Replogle R, Sheinin JC. Coronary artery bypass in a patient on lithium carbonate prophylaxis. J Nerv Ment Dis. 1982;170(3):181-4.

99. Blitt CD, Petty WC. Reversal of lorazepam delirium by physostigmine. Anesth Analg. 1975;54(5):607-8.

100. Galdston R. Psychotic reaction to the success of cardiac valvotomy: a case report. Psychiatry Med. 1970;1(4):367-73.
